# Host Response Impairs Tissue Integration of a Fibrin Hydrogel Scaffold Containing Poly(ε-caprolactone) Nanofibers for Peripheral Nerve Repair

**DOI:** 10.3390/biomimetics11090648

**Published:** 2026-09-09

**Authors:** Haktan Altinova, Dorothee Hodde, José L. Gerardo-Nava, Axel Dievernich, Lmar Arman, Melissa Büchler, Andreas Kriebel, Jörg Mey, Hans Clusmann, Joachim Weis, Gary A. Brook, Pascal Achenbach

**Affiliations:** 1 Institute of Neuropathology, Uniklinik RWTH Aachen, 52074 Aachen, Germany; 2 Department of Neurosurgery, Uniklinik RWTH Aachen, 52074 Aachen, Germany; 3 Division of Medical Assessment, State Office for Health and Social Affairs (Lageso), 10559 Berlin, Germany; 4 LMU University Hospital, LMU Medizin, Ludwig-Maximilians-Universität München, 80337 Munich, Germany; 5 DWI–Leibniz Institute for Interactive Materials, 52074 Aachen, Germany; 6 Institute of Applied Medical Engineering, Department of Advanced Materials for Biomedicine, Uniklinik RWTH Aachen, 52074 Aachen, Germany; 7 FEG Textiltechnik Forschungs- und Entwicklungsgesellschaft mbH, 52070 Aachen, Germany; 8 Department of Neurology, Uniklinik RWTH Aachen, 52074 Aachen, Germany; 9 Institute of Biology II, RWTH Aachen University, 52074 Aachen, Germany; 10 Laboratorio de Regeneración Neural, Hospital Nacional de Parapléjicos/IDISCAM, 45071 Toledo, Spain; 11 Phil and Penny Knight Campus for Accelerating Scientific Impact, University of Oregon, Eugene, OR 97403, USA

**Keywords:** host response, blood–nerve barrier, electrospinning, topography, guidance cues, biomimetic, bioengineering, tissue engineering

## Abstract

The development of bioengineered conduits for the repair of large peripheral nerve defects remains challenging. Here, we introduce a biomimetic fibrin hydrogel scaffold containing stacked arrays of aligned poly(ε-caprolactone) (PCL) nanofibers that mimics the naturally forming fibrin cable seen in transection injuries. We further evaluated the additional encapsulation of Schwann cells (SCs) into the fibrin hydrogel. Nerve regeneration was examined in a 15 mm rat sciatic nerve resection model over a period of 12 weeks, comparing our scaffolds against the autograft, a collagen hollow tube, and a lesion-only control group. Functional and morphometric analyses revealed that the autograft supported the strongest regeneration, followed by the SC-seeded fibrin-nanofiber scaffold and the hollow tube. Unexpectedly, a macrophage-rich core devoid of SCs and regenerated axons formed within both fibrin-nanofiber scaffolds around persisting PCL nanofibers. This core impaired the regenerative potential of the non-SC-seeded fibrin-nanofiber scaffold to the extent that functional regeneration was comparable to that of the lesion-only control group. While the scaffolds were designed to closely mimic and support the natural regenerative environment following nerve transection, our results reveal that these theoretical considerations do not necessarily translate into practice.

## 1. Introduction

Traumatic peripheral nerve injuries (PNIs) predominantly affect young, mostly male patients, and frequently result in long-term functional deficits, imposing a substantial socioeconomic burden [1]. In civilians, PNIs most commonly result from traffic accidents, circumscribed cuts or lacerations (e.g., from knives, glass, bites, machinery and tools), penetrating trauma (including gunshot and stab wounds), and iatrogenic causes [2,3,4,5,6].

Endogenous regeneration of a severed nerve critically depends on the integrity of its connective tissue layers—the endoneurium, perineurium, and epineurium—which provide guidance cues directing regenerating axons towards their distal targets [7]. When tensionless end-to-end coaptation is feasible, primary repair can leverage this intrinsic architecture. In complete transections with disruption to all connective tissue layers and segmental loss, however, a true gap prevents tensionless coaptation and makes repair particularly challenging. In such cases, an interponate is required to re-establish structural continuity and enable axon regrowth across the lesion site [8,9,10,11]. Clinically, the gold standard interponate is an autograft (AG) harvested from a healthy donor nerve [10,11,12]. AGs are known to provide orientational guidance via aligned basal lamina sheaths and a highly growth-permissive milieu due to resident repair-state Schwann cells (SCs) [10,11,13]. However, their use entails well-documented drawbacks [11,14] and outcomes remain unsatisfactory, with up to 50% of patients failing to achieve the desired extent of functional recovery [15].

These limitations have driven development of bioengineered alternatives to replace autografts in nerve repair, and several off-the-shelf conduits have been introduced [11]. Simple hollow tubes (HTs) made of collagen (multiple commercial products) or biodegradable polymers such as poly(glycolic acid) (NEUROTUBE^®^) are used for well-defined indications, typically short-gap repairs and purely sensory nerves [11]. Although such conduits have been shown to support regeneration across short distances, performance across critical-size gaps (>30 mm in humans, >10 mm in rats) remains poor [10,16,17], principally because they frequently fail to form a stable fibrin cable connecting the proximal and the distal nerve stumps [18,19].

Based on these findings, we developed a biomimetic nerve graft designed to support endogenous fibrin cable formation and provide topographical guidance for regenerative cell migration and axon outgrowth. We used a prefabricated collagen I/III HT [20,21,22] loaded with a fibrin hydrogel containing stacked arrays of aligned electrospun poly(ε-caprolactone) (PCL) nanofibers. This combined strategy distinguishes our scaffold design from previously published approaches aimed at inducing anisotropy directly within fibrin hydrogels [23]. PCL is a biodegradable polymer with U.S. Food and Drug Administration-approved clinical applications [24,25], including HTs for PNI repair (NEUROLAC^®^) [11]. To further enhance the pro-regenerative environment, nanofiber-containing fibrin hydrogels were optionally pre-seeded with isogenic SCs for direct delivery of repair-competent cells to the lesion site. Previously published work has already demonstrated that oriented PCL nanofibers can align SCs within fibrin hydrogels, a key step in forming aligned, growth-supportive pathways for regenerating axons that mimic bands of Büngner [26].

In this study, we evaluated fibrin-nanofiber scaffolds, both with (SC-FF) and without SCs (FF), in a rat model of severe PNI. Bioengineered conduits were implanted into a critical-size 15 mm sciatic nerve resection model and assessed over a period of 12 weeks. The regenerative potential of the implants was compared with the AG, a collagen HT, and a lesion-only (LO) control group using functional and morphometric analyses.

## 2. Materials and Methods

### 2.1. Electrospinning of Aligned PCL Nanofiber Arrays

Aligned PCL nanofiber arrays were produced by solution electrospinning using the gap method of alignment [23,24,25,26]. Briefly, an 8% (*w/v*) PCL (70–90 kDa, Sigma, Taufkirchen, Germany) solution in 3:1 (*v/v*) chloroform/methanol was pumped through a 21G nozzle (B. Braun, Melsungen, Germany) at 0.5 mL/h at an applied voltage of 12 kV. The suspended fibers were collected between the parallel bars (4 cm apart) of a custom V-shaped collector and subsequently transferred onto rectangular adhesive aluminum foil frames (inner size 30 × 23 mm).

### 2.2. Isolation and Culturing of Isogenic SCs

To prevent immune rejection after implantation of SC-loaded scaffolds, SCs were isolated from the sciatic nerves of isogenic 10-week-old female Lewis rats (Charles River Laboratories, Sulzfeld, Germany). SC isolation followed published protocols [23]: After removal of the epineurium, nerves were cut into small pieces and seeded into non-coated T25 dishes (Greiner Bio-One, Frickenhausen, Germany) containing high-glucose Dulbecco’s Modified Eagle Medium (DMEM, Life Technologies, Darmstadt, Germany) with 10% fetal bovine serum (FBS, GE Healthcare, Düsseldorf, Germany) and penicillin/streptomycin (10 U/mL and 10 mg/mL, respectively, GE Healthcare). The tissue fragments were cultured for seven days at 37 °C, 5% CO_2_ to allow fibroblasts to migrate out. Following this, the fragments were digested in 0.8% collagenase (Biochrom, Berlin, Germany) at 37 °C for two hours, followed by 30 min in 0.25% trypsin-EDTA (GE Healthcare). The dissociated cells were then seeded into poly-L-lysine (PLL)/laminin-coated (100 µg/mL and 10 µg/mL, respectively, Sigma) T75 flasks (Greiner Bio-One) and cultured in Schwann cell growth factor medium (SCGFM) containing high-glucose DMEM with 10% FBS, 50 µg/mL gentamicin (Sigma-Aldrich, Darmstadt, Germany), 100 µg/mL transferrin (Life Technologies), 8.3 ng/mL heregulin (R&D Systems, Minneapolis, MN, USA), 0.8 µM forskolin (Sigma), 8.3 ng/mL basic fibroblast growth factor (Life Technologies), and 10 µg/mL insulin (Sigma). Purified SC cultures (purity > 95% as revealed by immunofluorescence (IF)) were obtained by positive selection using a mouse monoclonal anti-nerve growth factor receptor/p75 antibody (1:500, MAB365, Millipore, Darmstadt, Germany) and the MiniMACS system (Miltenyi Biotec, Bergisch Gladbach, Germany), in accordance with the manufacturer’s protocol.

### 2.3. Manufacturing and Characterization of Fibrin-Nanofiber Scaffolds

The manufacture of nanofiber-containing fibrin hydrogels was performed as previously described [26]. To this end, ten adhesive aluminum foil frames with similarly aligned nanofiber arrays were stacked in 35 mm Petri dishes (Sarstedt, Nümbrecht, Germany) and sterilized by 30 min of UV exposure. A thrombin–calcium chloride solution in Tris-buffered saline was pipetted into the resulting molds. For SC-containing hydrogels, isogenic SCs were resuspended in this solution at 2 × 10^6^ cells/mL. Human fibrinogen was then added for 20 min at 37 °C to initiate gel polymerization, which yielded final thrombin, calcium chloride, and fibrinogen concentrations of 3 U/mL, 3.75 mM, and 5 mg/mL, respectively. After polymerization, nanofiber-containing fibrin hydrogels (±SCs) were released from the frames using a scalpel and transferred to Petri dishes containing SCGFM supplemented with tranexamic acid (160 µg/mL, Pfizer, Berlin, Germany) for one day prior to implantation. Immediately before implantation, hydrogels were cut into strips containing longitudinally oriented nanofibers and inserted into phosphate-buffered saline (PBS)-soaked, prefabricated type I/III collagen HTs (specifications according to manufacturer: inner diameter 1900 µm, wall thickness 50 µm, swelling rate 1.5–2) [20,21,22]. The strips were pulled into the conduits using a fine metal hook. The damaged end was cut off, and the strips were gently pushed back, leaving a protrusion of the conduit walls of a few millimeters on either side (Figure S1). Immediately following this, the conduits (±SCs) were implanted.

Mechanical testing assessed the effect of the embedded nanofiber arrays on fibrin hydrogel stiffness. The elastic modulus of cell-free hydrogels was measured using a DMA A800 dynamic mechanical analyzer (TA Instruments, New Castle, DE, USA). Hydrogels (±nanofibers) were cast in molds assembled from 30 stacked adhesive aluminum foil frames (inner size 18 × 18 mm, height 3 mm). The specimens remained in their molds and were compressed from above. The engineering elastic modulus was calculated for the 5–10% compression range.

Scanning electron microscopy revealed the microstructure of cell-free fibrin scaffolds containing stacked arrays of aligned PCL nanofibers. Scaffolds were fixed in 3.9% glutaraldehyde, dehydrated in acetone, critical-point dried (Polaron E3000, Quorum Technologies, Laughton, East Sussex, United Kingdom), bisected, mounted onto stubs, sputter-coated with gold, and imaged with an ESEM XL30 FEG (Philips, Eindhoven, The Netherlands) at 2 kV.

### 2.4. Animals and Experimental Groups

Female, 10-week-old Lewis rats (250–300 g) were housed in type IV macrolon cages with *ad libitum* access to water and a 12:12 h light-dark cycle. Even numbers of animals were randomly assigned to five groups (*n* = 12 per group): LO, AG, HT, FF and SC-FF. In the following, all groups except the LO group will be referred to as intervention groups. Two animals died during surgery (*n* = 1 in the LO group, *n* = 1 in the AG group), but all other animals recovered quickly and showed no signs of pain or other complications.

The majority of the animals (*n* = 7 in all groups) were finalized after 12 weeks. For early characterization of the scaffold composition and integrity following in vivo implantation (Figure S4), additional animals of the FF and SC-FF groups were finalized after two weeks. These animals were part of additional experiments not presented in this manuscript and, therefore, are not included in the *n* = 12 animals initially assigned to each group as described above. Animals that underwent retrograde tracing (see below) were finalized after 14 weeks (*n* = 5 in the HT, FF, and SC-FF groups; *n* = 4 in the LO and AG groups). Animals that were finalized after both 12 and 14 weeks contributed data to the behavioral analyses (i.e., grip strength analysis and static sciatic index). However, only animals that were finalized after 12 weeks contributed data to the muscle weight and histology-based analyses. Animals that were finalized after two weeks did not contribute data to this study other than that mentioned above. In addition to the animals lost during surgery, a number of animals from all the groups were excluded from some of the analyses (except for the grip strength analysis and the static sciatic index), mainly due to issues associated with tissue processing. Statistical analyses were performed only when sufficient numbers of animals from all groups were available. Where this criterion was not met, no statistical analyses were conducted (e.g., for the retrograde labelling of motor neurons).

### 2.5. Surgical Procedure

After brief isoflurane (Abbott GmbH, Wiesbaden, Germany) sedation, 0.03–0.05 mg/kg buprenorphine (Temgesic, Indivior Europe Ltd., Dublin, Ireland) was administered subcutaneously to prevent post-operative pain. Surgery was performed under isoflurane inhalation anesthesia (Model 400, Univentor High Precision Instruments, Zejtun, Malta) on a heating pad (Fine Scientific Tools, Heidelberg, Germany). Following incision of the skin, the right sciatic nerve was exposed by blunt dissection and transected distal to the sciatic notch and proximal to the trifurcation, creating a 15 mm gap.

In animals from the LO group, the proximal stump was sutured to the biceps femoris muscle to prevent spontaneous reconnection or neuroma formation. In AG-treated animals, the resected nerve segment was inverted, re-inserted and secured with individual tensionless 10-0 epineurial sutures (Ethicon Inc., Raritan, NJ, USA). Proximal and distal stumps were inserted ~1.5 mm into each end of the respective conduits in animals from the HT, FF, and SC-FF groups and secured with individual tensionless 10-0 epineurial sutures. Muscle and skin were closed in layers with 7-0 and 4-0 sutures, respectively.

### 2.6. Grip Strength Analysis

Motor recovery was assessed by measuring peak hind paw grip strength using a dedicated test device (Bioseb, Vitrolles Cedex, France). Rats were trained to grasp a metal grid attached to the sensor and were then gently drawn backward until the hind paw grip was released or slipped. Peak force was recorded for three trials per animal and time point. Measurements were obtained pre-operatively and weekly until 12 weeks after surgery.

### 2.7. Static Sciatic Index (SSI)

Hind paw motor function under static conditions with weight support was evaluated using the “Visual-SSI” as described before [27] with minor modifications. A custom-made MATLAB (version 8.1, The MathWorks, Inc., Natick, MA, USA) code was used to automate measurements of the relevant parameters and execute calculations using the established equations. Measurements were obtained pre-operatively and tri-weekly until 12 weeks after surgery.

### 2.8. Tissue Processing and Muscle Weight Measurement

All animals were euthanized by terminal anesthesia and transcardially perfused with 0.1 M PBS, followed by 4% paraformaldehyde (PFA) in PBS.

Only the sciatic nerves from both sides were harvested from animals finalized after two weeks. For animals finalized after 12 weeks, the sciatic nerves and hindlimb muscles—extensor digitorum longus (EDL), tibialis anterior (TA), soleus (SOL) and gastrocnemius (GC)—were harvested from the lesioned and contralateral sides. For animals finalized after 14 weeks, L3-L6 levels of the spinal cord and L4/L5-level dorsal root ganglia (DRGs) were retrieved.

Sciatic nerve explants were photographed using a stereomicroscope equipped with an AxioCam digital camera (Zeiss, Oberkochen, Germany), postfixed for 24 h in 4% PFA, washed in PBS, and stored in PBS containing 0.05% NaN_3_ at 4 °C until further use. The same post-fixation and storage procedure was also applied to the spinal cords. DRGs, in contrast, were dehydrated through a series of graded alcohols (70%, 96% and 100% ethanol, each for five minutes), infiltrated with xylene and paraffin-embedded.

The muscles were dried and weighed on a precision scale (A120S, Sartorius AG, Göttingen, Germany). The lesioned-to-contralateral muscle weight ratio was then calculated.

### 2.9. Retrograde Tracing

Retrograde tracing using AlexaFluor 594-conjugated Dextran (10 kDa, Life Technologies) was performed to identify the cell bodies of spinal cord motor and DRG sensory neurons, whose axons had successfully regenerated across the sciatic nerve resection injury, providing a measure of motor and sensory nerve fiber regeneration, respectively. The repaired sciatic nerves were re-exposed 12 weeks after initial surgery and the tibial, peroneal and sural nerves were then transected 5–8 mm distally to the trifurcation. Crystals of the tracing reagent were applied for 30 min. Following thorough washing of the nerve stumps and closure of muscle and skin, the animals were allowed to survive for another two weeks for the tracers to be retrogradely transported towards the motor and sensory neuron cell bodies.

Following finalization, tracer detection by means of fluorescence microscopy was performed on 40 µm thick sections of the spinal cord (L3-L6 levels, every third section used for quantification) and 5 µm thick sections of the L4/L5 DRGs (every third section used for quantification), counterstained with 4′,6-diamidino-2-phenylindole (DAPI). The absolute numbers of labeled motor and sensory neurons were collected. For more detailed information on the tissue processing and DAPI staining prior to fluorescence microscopy, see below (as described in more detail for the sciatic nerve explants). Staining of DRG tissue sections was performed only following de-waxing and re-hydration. For this, the paraffin-embedded sections were incubated in xylene for 20 min followed by sequential incubation in decreasing concentrations of alcohol (100%, 96% and 70% ethanol, each for five minutes) and three washes with 0.1 M PBS.

### 2.10. Hematoxylin/Eosin (HE) Staining and IF

PFA-fixed sciatic nerve explants were cryoprotected in 30% sucrose in PBS at 4 °C for 48 h, embedded in Tissue-Tek O.C.T. Compound (Sakura, Umkirch, Germany), and snap-frozen in isopentane at −80 °C. Samples proximal and distal to the lesion site and at the mid-implant level were cut into 15 µm cross-sections and collected onto object slides (SuperFrost Ultra Plus, Menzel-Gläser, Braunschweig, Germany).

For HE staining, sections were incubated in Mayer’s hemalum solution (Sigma-Aldrich) for ten minutes, rinsed in water, and stained with 1% eosin (Carl Roth, Karlsruhe, Germany) for five minutes. Sections were then dehydrated in a graded ethanol series (70%, 96%, 100%), cleared in xylol, and coverslipped with Vitro-Clud (R. Langenbrinck, Emmendingen, Germany). Images were acquired using a GRYPHAX SUBRA digital camera (Jenoptik, Jena, Germany) on an AxioScope A1 microscope (Zeiss) equipped with a polarized light filter.

Double IF was performed using a number of primary antibody combinations (Table 1). Sections were incubated in 1:1 (*v/v*) acetone/methanol for 20 min at 4 °C, then blocked for one hour at room temperature in PBS containing 10% normal goat serum (Sigma-Aldrich) and 0.5% Triton X-100 (Sigma-Aldrich). Primary antibodies, diluted in the same buffer, were incubated overnight at room temperature. Secondary antibodies Alexa Fluor 488 goat anti-mouse IgG (A11029, Invitrogen, Darmstadt, Germany), Alexa Fluor 594 goat anti-mouse IgG (A11032, Invitrogen), Alexa Fluor 488 goat anti-rabbit IgG (A11034, Invitrogen), and Alexa Fluor 594 goat anti-rabbit IgG (A11037, Invitrogen) were diluted 1:500 in PBS and incubated for two hours at room temperature. Nuclei were counterstained with DAPI (1:1000, 62248, ThermoFisher, Darmstadt, Germany) during secondary antibody incubation. For rat endothelial cell antigen 1 (RECA1) and prolyl-4-hydroxylase β (P4Hβ), biotin-streptavidin signal enhancement was employed: After primary antibody incubation, sections were incubated in a biotinylated goat anti-mouse antibody (1:500, BA-9200, Vector Laboratories, Newark, CA, USA) for one hour at room temperature, washed in PBS, and incubated in Alexa Fluor 488-conjugated streptavidin (1:500, S32354, Invitrogen) or Alexa Fluor 594-conjugated streptavidin (1:500, S32356, Invitrogen) for two hours at room temperature. Sections were cover-slipped with fluorescence mounting medium (S3023, Dako, Waldbronn, Germany) and imaged using either a BX51 fluorescence microscope (Olympus, Hamburg, Germany) with a DP72 digital camera (Olympus) or an Axioplan microscope (Zeiss) with an AxioCam MRc digital camera (Zeiss).

### 2.11. Image Processing and Quantification

Image processing and quantification were performed using FIJI for macOS (version 2.9.0/1.53t). Macroscopic images of nerve explants were combined with the built-in stitching function. Cross-section areas (CSAs) of nerve explants at the mid-implant level were measured in low-magnification images of TUBB3/S100-stained sections by manually tracing nerve outlines with the selection tool. For quantification of TUBB3-positive pixels at the mid-implant level and in the distal nerve, outlines of the nerve were also marked manually. Following this, the TUBB3 channel was isolated, contrast and brightness were adjusted, and binary images were generated to determine the number of TUBB3-positive pixels, which was then related to the CSA. These steps were supported by a custom-made FIJI script. GLUT1-positive pixels at mid-implant level were quantified in the same way. Mini-fascicle size was quantified using the GLUT1 signal. Signal isolation, adjustment of brightness and contrast, and transformation into a binary image were carried out, followed by the application of the watershed algorithm. Individual mini-fascicles were selected with the wand tool to calculate their CSAs. These steps were also supported by a custom-made FIJI script. For retrograde labelling, only neuronal profiles exhibiting a clear signal and containing a DAPI-labelled nucleus within the ventral horn of the spinal cord or within the DRG were counted as positive.

### 2.12. Statistical Analyses

Statistical analyses were performed using GraphPad Prism 11.0.0 for macOS. Data sets and statistical tests are specified in the figure legends; *p*-values <0.05 were considered statistically significant. Statistical significance is indicated as * *p* < 0.05, ** *p* < 0.01, *** *p* < 0.001, **** *p* < 0.0001. Data throughout the main text of the manuscript are reported as mean ± standard error of the mean (SEM), unless otherwise stated.

### 2.13. Use of Generative Artificial Intelligence

Generative artificial intelligence was employed for the purpose of language editing (RWTHgpt, KI:connect.nrw, RWTH Aachen, Aachen, Germany) as well as for the generation of schematics for the graphical abstract (Gemini 3.1 Pro, Google LLC, Mountain View, CA, USA).

## 3. Results

### 3.1. Characterization of FF Scaffolds

**Figure 1:**
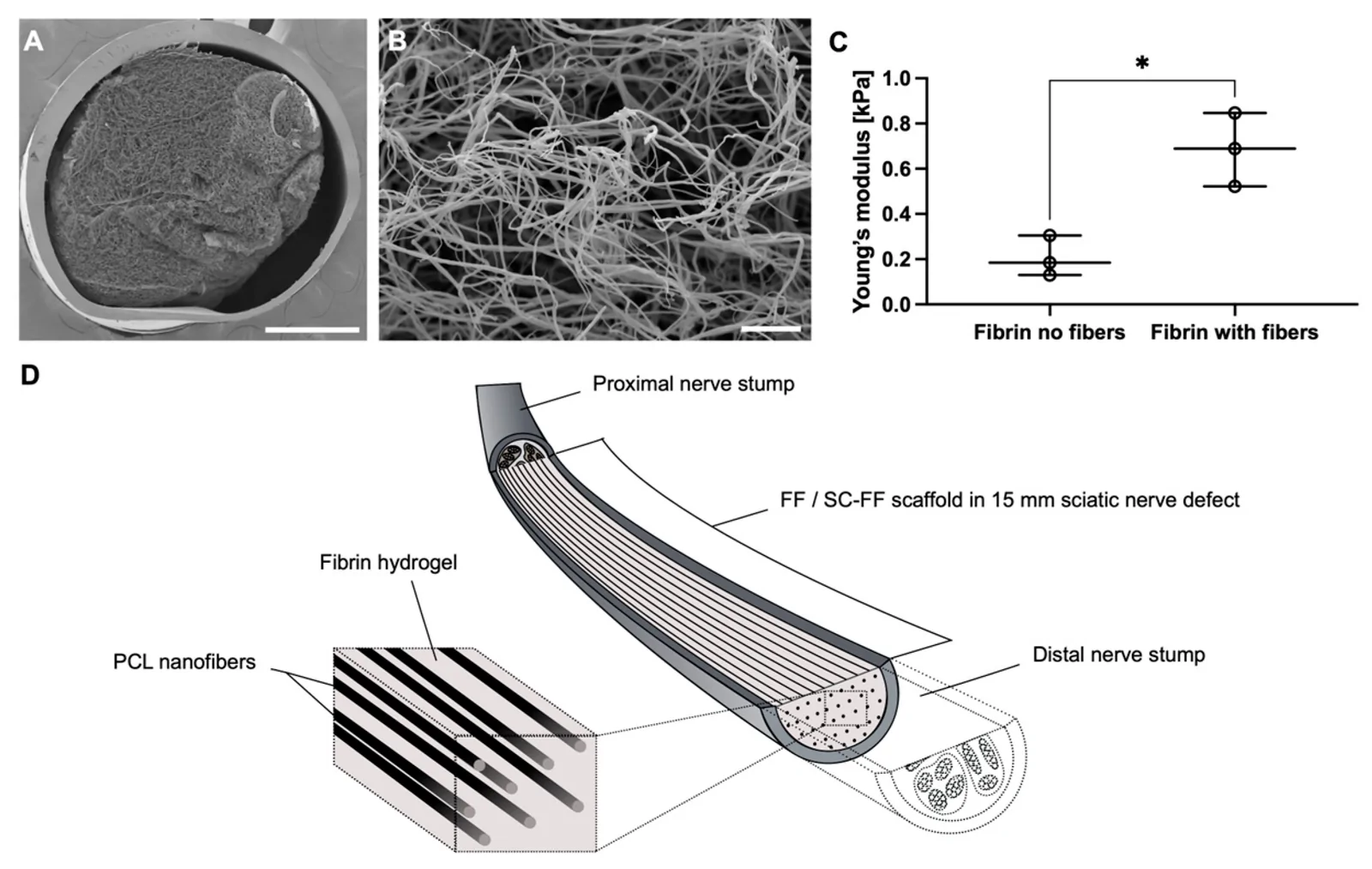
Characterization of the fibrin-nanofiber scaffold. (**A**) Scanning electron microscopy cross-section at the mid-implant level of a prefabricated collagen hollow tube (HT) filled with the nanofiber-containing fibrin hydrogel. (**B**) Close-up of the fibrin-nanofiber hydrogel reveals that the poly(ε-caprolactone) (PCL) nanofibers are indistinguishable from fibrin fibrils. (**C**) Statistical analysis of the elastic moduli of fibrin hydrogels with and without embedded PCL nanofiber arrays. (**D**) Schematic drawing of the scaffold depicting the intended distribution of PCL nanofibers within the fibrin hydrogel as well as the intended integration of the conduit into the lesion site following nerve repair. Scale bars: (**A**) 500 µm, (**B**) 5 µm. Data in (**C**) are presented as boxplots showing individual values, their spread, and the 1st, 2nd and 3rd quartiles. Statistical significance was assessed using an unpaired t-test with Welch’s correction (* *p* < 0.05, *n* = 3 samples for each condition).

Scanning electron microscopy revealed that the PCL nanofiber-loaded fibrin hydrogel almost completely filled the lumen of the prefabricated collagen HT (Figure 1A). Nanofibers were indistinguishable from fibrin fibrils (Figure 1B), which is in line with the sub-micron diameters reported earlier for fibers generated under similar conditions [26]. Previous results showed an average spacing of ~50 µm between similarly stacked nanofiber arrays, with about 80% of fibers being aligned within ±10° of the main axis [26]. Mechanical testing (Figure 1C) revealed a slight but significant increase in elastic modulus for nanofiber-containing hydrogels (0.69 ± 0.09 vs. 0.21 ± 0.05 kPa). Figure 1D provides a schematic overview of the anticipated scaffold composition (highlighting the intended distribution of PCL nanofiber arrays within the fibrin hydrogel) and tissue integration following in vivo implantation.

### 3.2. Functional Analyses and Retrograde Tracing

**Figure 2:**
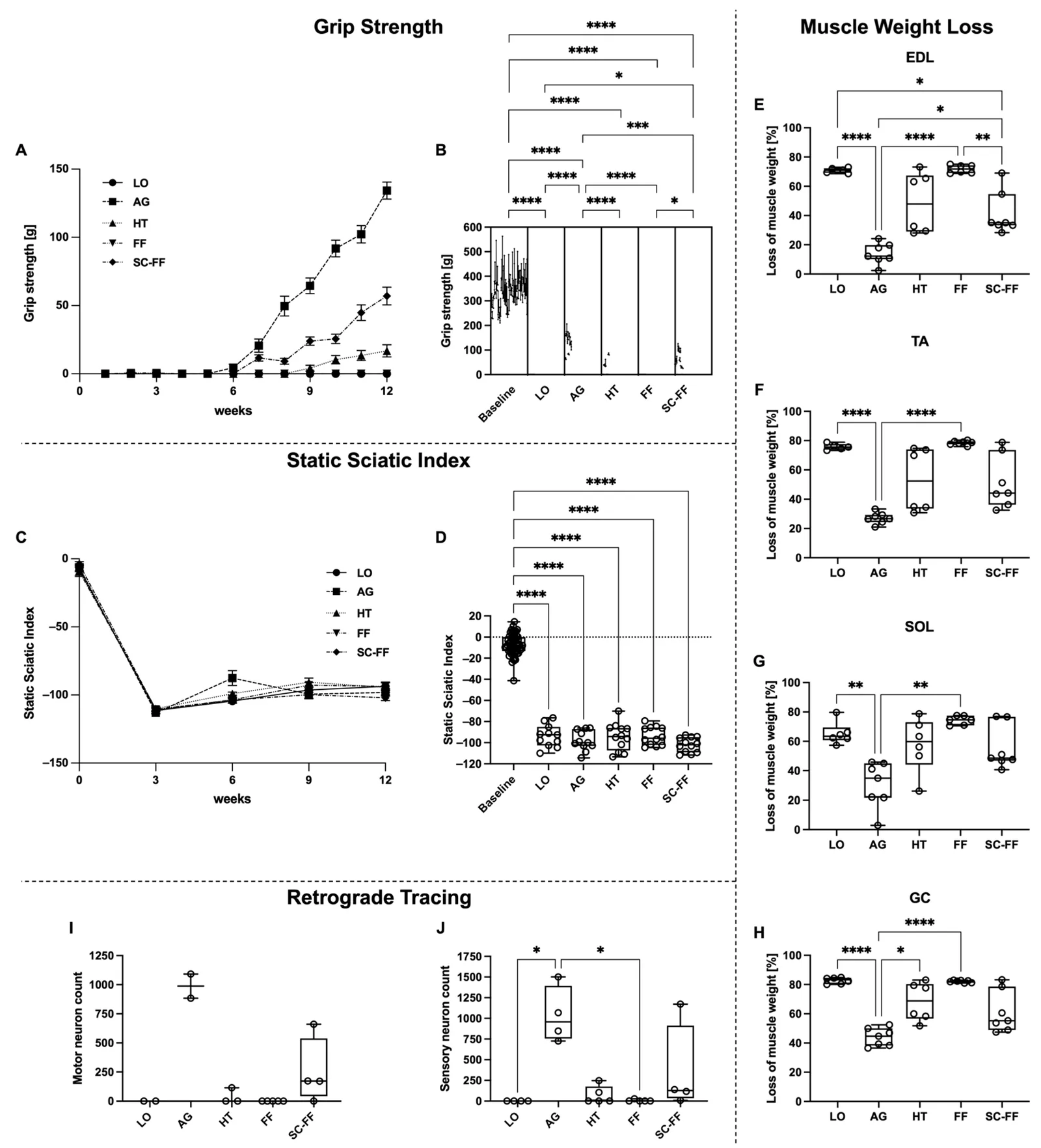
Results of the behavioral analyses and retrograde tracing of motor and sensory nerve fibers after sciatic nerve repair in all intervention groups and the lesion-only control group. (**A**,**B**) Hind paw grip strength. Results from weekly measurements shown in ((**A**), data points in the LO and FF groups overlap, as well as with the x-axis as no recovery was observed); statistical analysis of the final values obtained after 12 weeks shown in (**B**). (**C**,**D**) Static sciatic index (SSI). Results from tri-weekly measurements shown in (**C**); statistical analysis of the final values obtained after 12 weeks shown in (**D**). (**E**–**H**) Quantification and statistical analysis of muscle weight loss after 12 weeks for the extensor digitorum longus muscle (EDL) in (**E**), tibialis anterior muscle (TA) in (**F**), soleus muscle (SOL) in (**G**), and gastrocnemius muscle (GC) in (**H**). (**I**,**J**) Results of retrograde tracing of motor (**I**) and sensory nerve fibers (**J**) 14 weeks after sciatic nerve repair. Data are presented as mean ± standard error of the mean in (**A**,**C**) or as boxplots in (**B**,**D**–**J**) indicating individual values (in (**B**) per animal as multiple data points were obtained from one animal), their spread as well as the 1st, 2nd and 3rd quartiles. Statistical significance for grip strength measurements was tested using a nested one-way analysis of variance (ANOVA) followed by a Tukey test for multiple comparisons between pairs of means (* *p* < 0.05, *** *p* < 0.001, **** *p* < 0.0001; *n* = 174 measurements for baseline from 58 animals, *n* = 33 measurements in the autograft (AG) group from 11 animals, *n* = 33 measurements in the lesion-only (LO) group from 11 animals, *n* = 36 measurements in the HT, fibrin-nanofiber scaffold (FF), and Schwann cell-seeded fibrin-nanofiber scaffold (SC-FF) groups from 12 animals in each group). Statistical significance for SSI was tested using an ordinary one-way ANOVA followed by a Holm–Šidák multiple comparisons test with a single pooled variance for multiple comparisons between pairs of means (**** *p* < 0.0001; *n* = 58 animals for baseline, *n* = 11 animals in the LO and AG groups, *n* = 12 animals in the HT, FF, and SC-FF groups). Statistical significance for muscle weight loss was tested using Brown–Forsythe and Welch’s ANOVA tests followed by a Dunnett’s T3 post-hoc test for multiple comparisons between pairs of means (* *p* < 0.05, ** *p* < 0.01, **** *p* < 0.0001; for EDL and SOL: *n* = 6 animals in the LO, HT, and FF groups, *n* = 7 animals in the AG and SC-FF groups; for TA: *n* = 5 animals in the LO group, *n* = 6 animals in the HT group, *n* = 7 animals in the AG, FF, and SC-FF groups; for GC: *n* = 6 animals in the LO and HT groups, *n* = 7 animals in the AG, FF, and SC-FF groups). No statistical tests were performed for motor neuron count due to the small sample size in some of the groups (*n* = 2 animals in the AG and LO groups, *n* = 3 animals in the HT group, *n* = 4 animals in the SC-FF group, *n* = 5 animals in the FF group). Statistical significance for sensory neuron count was tested using a Kruskal–Wallis test followed by a Dunn’s post-hoc test for multiple comparisons between pairs of means (* *p* < 0.05; *n* = 4 animals in the AG, LO, and SC-FF groups, *n* = 5 animals in the HT and FF groups).

Following surgery, hind paw grip strength was measured weekly over 12 weeks to assess functional recovery (Figure 2A,B). The pooled pre-operative baseline strength including all animals across all groups was 342.89 ± 5.09 g. No recovery was seen in any group until week six, by which time grip strength began to gradually increase in the AG group (4.51 ± 2.54 g), followed by the SC-FF group at week seven (11.67 ± 2.39 g) and the HT group at week nine (4.11 ± 2.15 g). Grip strength continued to rise without reaching a plateau in all these groups, with final 12-week values of 134.34 ± 6.20 g in the AG group, 57.02 ± 6.44 g in the SC-FF group, and 16.88 ± 4.48 g in the HT group. No recovery was observed in either the LO or FF groups (Figure 2A). By week 12, no group had reached baseline performance. The AG group performed significantly better than all other groups; however, grip strength in the SC-FF group significantly exceeded both the LO and FF groups. No significant differences were found between any of the other groups. (Figure 2B).

The SSI was measured at three-week intervals over the 12-week follow-up period after surgery. Only slight improvements following almost the same trends were observed across all groups, revealing that a practically plateau-like phase had already been reached between the nine- and 12-week time points (Figure 2C). No significant differences were observed between any of the groups after 12 weeks. However, all groups exhibited significantly lower SSI values than the pre-operative baseline value of −7.06 ± 1.28 pooled from all animals across all groups (Figure 2D). By this time, the SSI had reached final values of −93.65 ± 3.16 in the LO group, −98.14 ± 2.84 in the AG group, −94.45 ± 3.70 in the HT group, −93.68 ± 2.53 in the FF group and −102.07 ± 2.00 in the SC-FF group.

Muscle atrophy was assessed by comparing the weight of the EDL, TA, SOL, GC on the lesioned side to the contralateral side (Figure 2E–H). The AG group showed the least muscle weight loss, which was significantly less than in both the LO and FF groups for all muscles. Intermediate levels of muscle weight loss were observed in the HT and SC-FF groups, with no significant differences between the two. Significant differences were found between the AG group and the SC-FF group for the EDL and between the AG group and the HT group for the GC. Significant differences between the SC-FF group and the FF or LO groups were only observed for the EDL. Mean values of muscle weight loss ± SEM (in % compared with the contralateral side) are contained in Table S1 for each muscle and group.

Retrograde tracing of spinal cord motor and DRG sensory neurons was performed to correlate the outcomes of the behavioral and muscle weight loss analyses with the extent of motor fiber regeneration and to further assess the extent of sensory nerve fiber regeneration. Data on retrogradely labeled motor neurons could only be obtained from *n* = 2 animals in the LO and AG groups due to technical issues. This meant that no statistical evaluation was feasible and that these preliminary data must be interpreted with caution. Despite these limitations, however, the data revealed a trend toward the number of labeled motor neurons being highest in the AG group (988.00 ± 104.00), followed by the SC-FF and HT groups (251.25 ± 142.47 and 38.33 ± 38.33, respectively). No labeled spinal cord motor neurons were seen in either the FF or LO groups. Consistent with these trends, the number of retrogradely labeled sensory neurons was highest in the AG group (1035.75 ± 170.78), followed by the SC-FF and HT groups (358.50 ± 272.65 and 70.20 ± 48.57, respectively). Very few labeled sensory neurons were found in the FF group (5.80 ± 5.55), with none in the LO group. Statistical analysis revealed that whilst the number of sensory neurons was significantly greater in the AG group in comparison with both the FF and LO groups, no statistically significant differences were observed between any of the other groups.

### 3.3. Macroscopic Examination of Sciatic Nerve Explants

**Figure 3:**
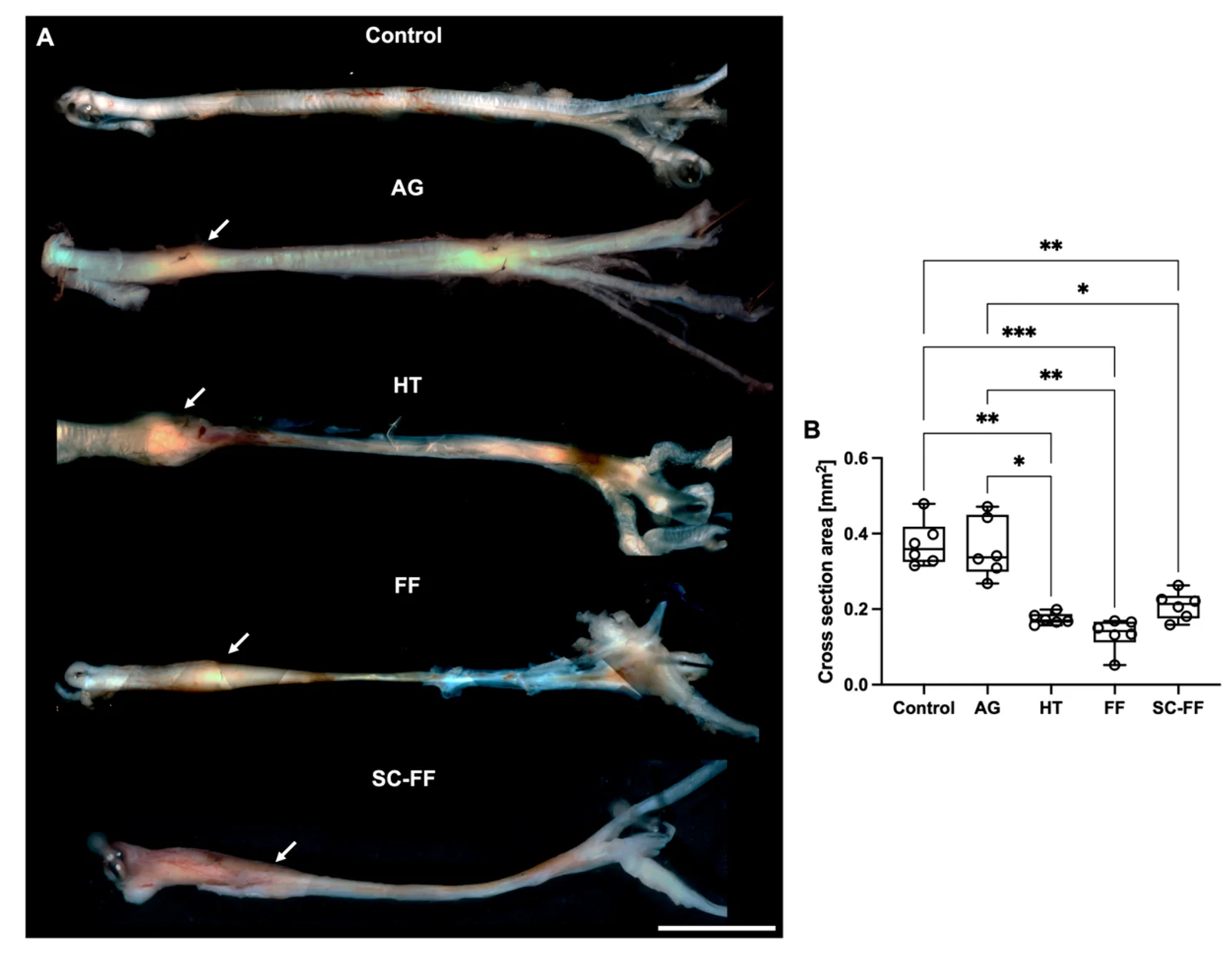
Macroscopic appearance of nerve explants and cross-section area (CSA) quantification at the mid-implant level in all intervention groups. (**A**) Overview of nerve explants. Arrows indicate swelling at the proximal coaptation site. The AG is almost indistinguishable from the non-lesioned control nerve. In all other groups, tissue cables of varying diameters connecting the proximal and distal nerve stumps have formed, irrespective of the type of intervention. No remnants of the collagen scaffolds or nanofiber-containing fibrin hydrogels are visible. (**B**) Quantification and statistical analysis of the mid-implant CSAs based on data collected from histological sections. Scale bar: 5 mm. Data are presented as boxplots indicating individual values, their spread, as well as the 1st, 2nd and 3rd quartiles. Statistical significance was tested using Brown–Forsythe and Welch’s ANOVA tests followed by a Dunnett’s T3 post-hoc test for multiple comparisons between pairs of means (* *p* < 0.05, ** *p* < 0.01, *** *p* < 0.001; *n* = 6 animals in all groups).

Twelve weeks following sciatic nerve injury, the repaired nerves—including the adjacent, spared proximal and distal nerve segments—were excised and inspected, providing a general impression of the extent of tissue regeneration using the different intervention strategies. Macroscopically (Figure 3A), AG explants were found to be almost indistinguishable from control nerves. HT, FF and SC-FF explants all showed regenerated tissue cables connecting the proximal and distal nerve segments, although these were of varying diameters. Swelling was common near the proximal anastomosis site. All collagen conduits implanted in the HT, FF, and SC-FF groups were completely degraded. Similarly, no remnants of the PCL nanofiber-containing fibrin hydrogels were observed macroscopically. Histological quantification of the mean CSA at the mid-implant level (Figure 3B) revealed no significant differences between the AG group (0.36 ± 0.03 mm^2^) and control nerves (0.37 ± 0.02 mm^2^). However, both groups demonstrated significantly greater CSAs than the HT, FF, and SC-FF groups (0.17 ± 0.01 mm^2^, 0.13 ± 0.02 mm^2^ and 0.21 ± 0.02 mm^2^, respectively), which did not differ significantly from one another.

### 3.4. Histological Characterization of Sciatic Nerve Explants

Detailed immunohistochemical analyses of the sciatic nerve explants were performed to characterize and compare the tissue response as well as the extent of regeneration between the different intervention groups.

**Figure 4:**
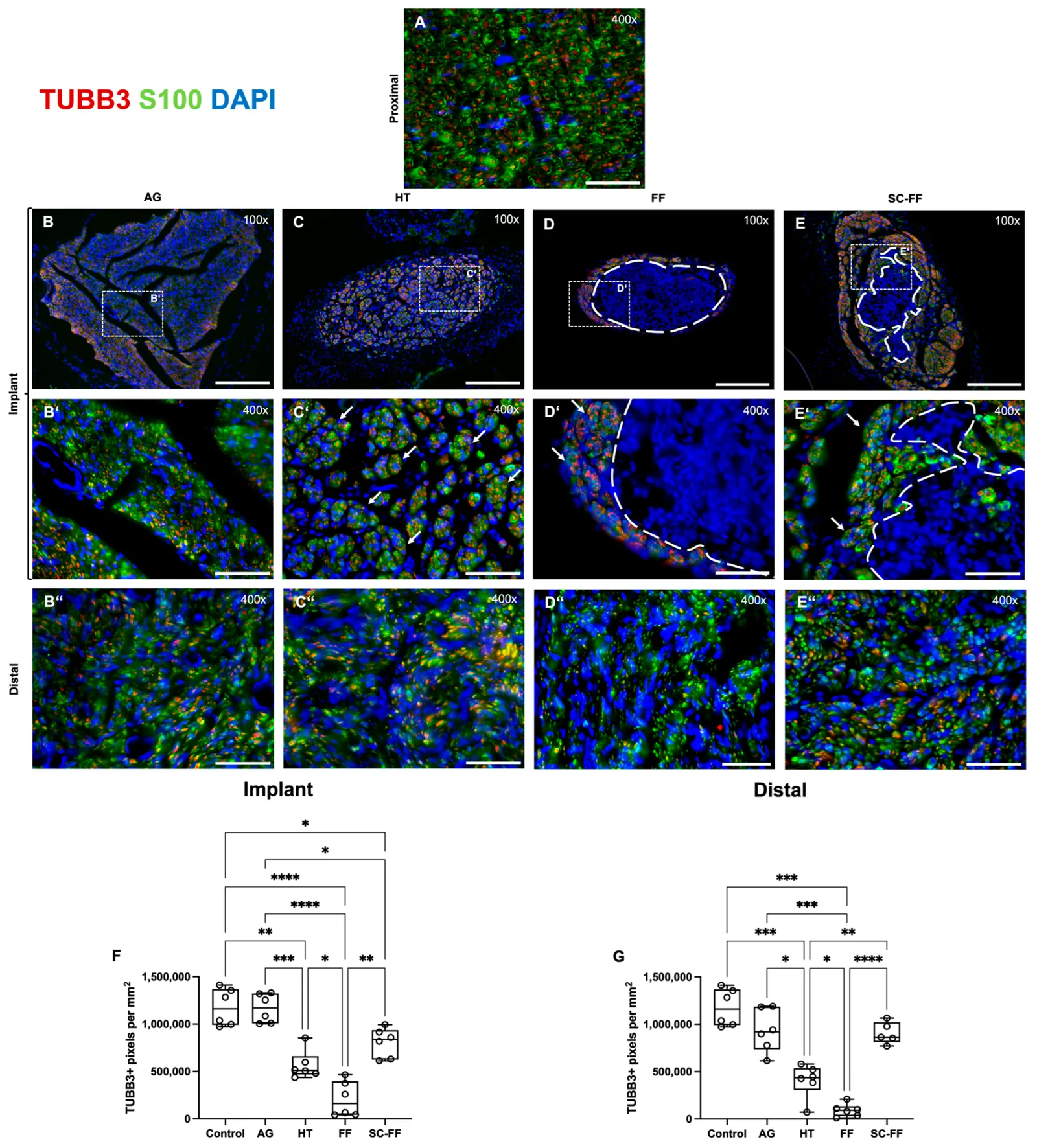
βIII-tubulin (TUBB3) and protein S100β (S100) immunofluorescence (IF) of sciatic nerve explants from all intervention groups (cross-sections). (**A**) Representative image of the proximal nerve segment in all groups showing Schwann cells (SCs, labelled by S100 in green) and axons (labelled by TUBB3 in red). (**B**–**B’’**) In the AG group, numerous SCs and regenerated axons can be found at the mid-implant level (**B**,**B’**) and in the distal segment (**B’’**). In direct comparison, distal axons appear smaller in diameter. 4′,6-diamidino-2-phenylindole (DAPI) staining (nuclei, blue) highlights the increased cellularity at the mid-implant and distal levels in comparison with the proximal segment. (**C**–**C’’**) Groups of regenerated mini-fascicles at the mid-implant level in the HT group ((**C**,**C’**), arrows in (**C’**)). Distal SC and axon density appear lower than in the AG group (**C’’**). Cell density appears higher in the distal nerve segment (**C’’**) than in the proximal part. (**D**–**D’’**) A cell-dense core devoid of SCs and axons (dashed outlines in (**D**,**D’**)) with only a few regenerated mini-fascicles (arrows in (**D’**)) can be seen at the mid-implant level in the FF group. Distally (**D’’**), only sporadic small-diameter regenerated axons with accompanying SCs are visible, while cellularity appears increased compared with the proximal nerve segment. (**E**–**E’’**) A similar core (dashed outline in (**E**,**E’**)) but with markedly more regenerated mini-fascicles around and within can be seen in the SC-FF group ((**E**,**E’**), arrows in (**E’**)). Distal SC and axon density appear comparable to the HT group but lower than in the AG group (**E’’**). Cell density within the distal nerve segment (**E’’**) appears higher compared with the proximal one. (**F**,**G**) Quantification and statistical analysis of the number of TUBB3-positive pixels per mm^2^ at the mid-implant level (**F**) and within the distal nerve segment (**G**) support the qualitative observations. Scale bars: (**B**,**C**,**D**,**E**) 200 µm; others 50 µm. Magnification is indicated in the upper right corner for all images. Dashed boxes in the low-power images indicate the region shown at higher magnification in the corresponding high-power images. Data are presented as box plots in (**F**,**G**), indicating individual values, their spread, as well as the 1st, 2nd and 3rd quartiles. Statistical significance was tested using Brown–Forsythe and Welch’s ANOVA tests followed by a Dunnett’s T3 post-hoc test for multiple comparisons between pairs of means (* *p* < 0.05, ** *p* < 0.01, *** *p* < 0.001, **** *p* < 0.0001; *n* = 6 animals in all groups except the SC-FF group at distal level, *n* = 5 animals in the SC-FF group at distal level).

SCs and regenerated axons were visualized using antibodies against S100 and TUBB3 (Figure 4). In cross-sections, nerve architecture in the proximal nerve across all groups (representative image shown in Figure 4A) was similar to non-lesioned control nerves, except an overall higher cell density was observed (see below). The mid-implant sections derived from the AG group resembled proximal nerves but demonstrated increased cellularity (Figure 4B,B’). Numerous SCs and axons were identified within the graft and the distal segment, far beyond the distal anastomosis. Furthermore, the distal nerve segment also showed increased cellularity when compared with the proximal nerve segment (Figure 4B’’). Regenerated axons at the mid-implant level and within the distal part appeared to be smaller than those within the proximal nerve segment. Similar observations were made in the HT, FF, and SC-FF groups, with the distal nerve segment exhibiting a higher cell density in comparison with the proximal segment and the axons in the distal nerve segment appearing to be of smaller diameter (Figure 4C’’–E’’). The distribution of SCs and axons within the tissue bridge of the HT group resembled mini-fascicles (Figure 4C,C’). Interestingly, both the FF and SC-FF groups consistently showed a cell-rich core that was practically S100- and TUBB3-negative and located at the center of the tissue bridges at the mid-implant level (Figure 4D,D’,E,E’). This core was surrounded by varying numbers of regenerated mini-fascicles, more abundant in the SC-FF than in the FF group (Figure 4D,D’,E,E’). Quantification of the number of TUBB3-positive pixels at the mid-implant level and distally was performed to quantify the success of axon regeneration across the proximal and distal suture sites. Data acquired from both sites were compared between groups and against a non-lesioned control nerve (1,176,273 ± 80,124). Quantification at the mid-implant level (Figure 4F) revealed no significant differences between the AG group (1,167,088 ± 62,272) and control nerves, with both groups demonstrating significantly greater values than all other groups. The HT and SC-FF groups (565,102 ± 61,687 and 804,813 ± 62,825, respectively) both exceeded the FF group (208,271 ± 75,682) with no significant difference between the two. Distally (Figure 4G), axon counts did not differ significantly for control nerves, the AG group (934,748 ± 92,350), and the SC-FF group (906,637 ± 51,500), all being greater than the HT and FF groups (405,086 ± 72,647 and 91,467 ± 28,044, respectively). The FF group revealed the presence of significantly fewer TUBB3-positive pixels than all other groups.

**Figure 5:**
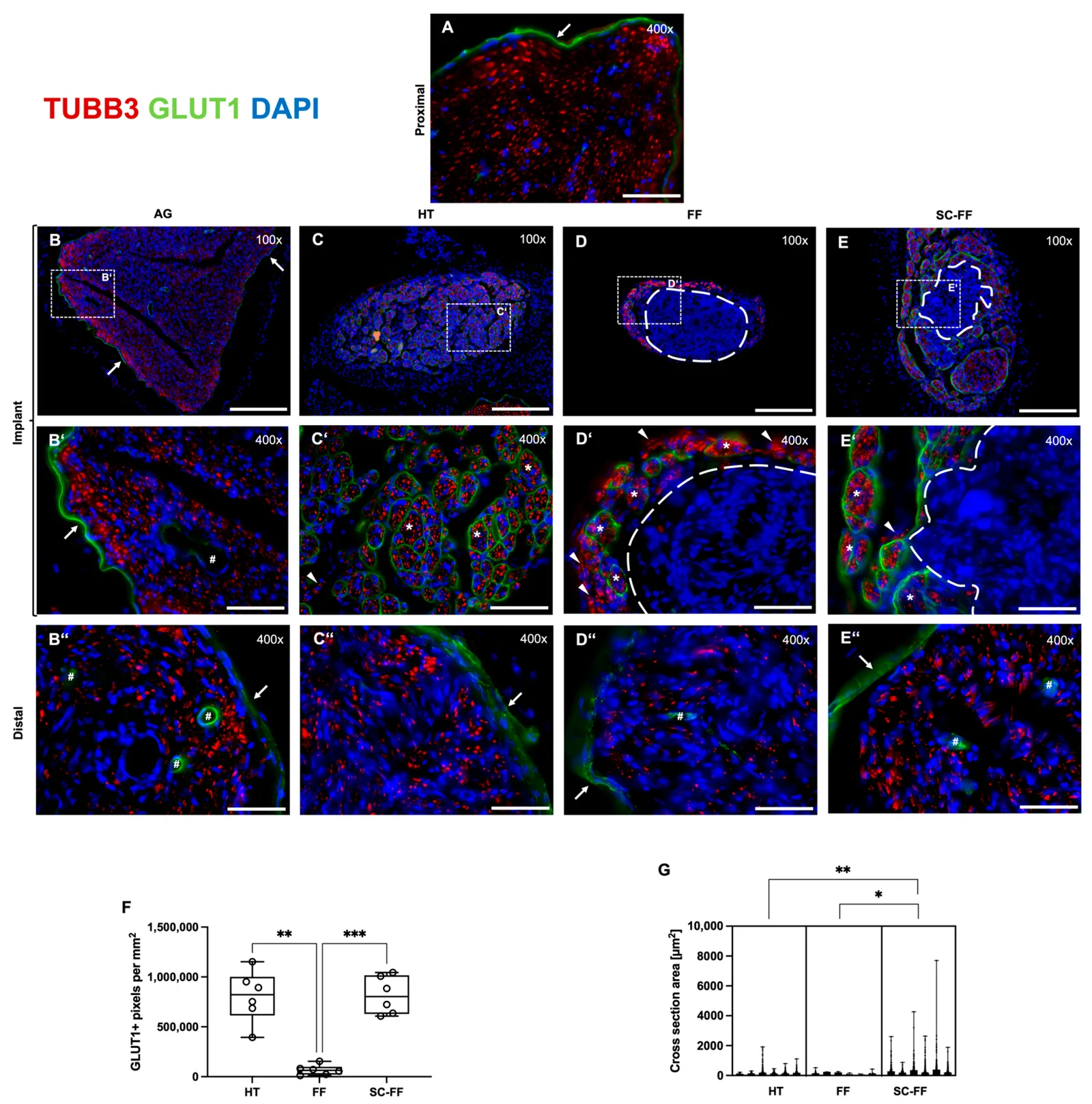
TUBB3 and glucose transporter 1 (GLUT1) IF of sciatic nerve explants from all intervention groups (cross-sections). (**A**) Proximal nerve segment representative of all groups with numerous axons (red). The fascicle is surrounded by a continuous GLUT1-positive perineurium (green, arrow). (**B**–**B’’**) At the mid-implant level ((**B**), higher magnification in (**B’**)) and in the distal segment (**B’’**), the AG resembles the architecture of the proximal nerve segment, demonstrating a continuous perineurium (arrows). Blood vessels with GLUT1-positive endothelial cells are indicated by hashtags in (**B’**,**B’’**). (**C**–**C’’**) Mini-fascicles at the mid-implant level in the HT group are individually wrapped by a thin perineurial cell (PNC) layer ((**C**,**C’**), asterisks in (**C’**)). Only a few axons lack perineurial ensheathment (arrowhead in (**C’**)). The distal nerve segment shows the same tissue organization as proximally ((**C’’**), perineurium indicated by arrow). (**D**–**D’’**) A few PNC-surrounded mini-fascicles (asterisks in (**D’**)) can be seen around the central core (dashed lines in (**D**,**D’**)) at the mid-implant level in the FF group, with numerous regenerated axons lacking perineurial ensheathment (arrowheads in (**D’**)). The distal nerve segment exhibits an organization similar to that of the proximal part ((**D’’**), perineurium indicated by arrow). A blood vessel with GLUT1-positive endothelial cells is indicated by a hashtag (**D’’**). (**E**–**E’’**) The core region in the SC-FF group (dashed lines in (**E**,**E’**)) is surrounded by numerous mini-fascicles enveloped by PNCs (asterisks in (**E’**)). In some areas, the perineurium forms an almost continuous sheath around the core. Only individual regenerated axons lack perineurial ensheathment (arrowheads in (**E’**)). As in the FF group, the distal part resembles the proximal nerve segment ((**E’’**), perineurium indicated by arrow). Blood vessels with GLUT1-positive endothelial cells are indicated by hashtags in (**E’’**). (**F**,**G**) Quantification and statistical analysis of the number of GLUT1-positive pixels at the mid-implant level (**F**) and the CSAs of mini-fascicles (**G**), both supporting the qualitative observations. Scale bars: (**B**,**C**,**D**,**E**), 200 µm; others, 50 µm. Magnification is indicated in the upper right corner for all images. Dashed boxes in the low-power images indicate the region shown at higher magnification in the corresponding high-power images. Data are presented as boxplots in (**F**,**G**) indicating individual values (in (**G**) per animal, as multiple data points were obtained from one animal), their spread, as well as the 1st, 2nd and 3rd quartiles. Statistical significance in (**F**) was tested using Brown–Forsythe and Welch’s ANOVA tests followed by a Dunnett’s T3 post-hoc test for multiple comparisons between pairs of means (** *p* < 0.01, *** *p* < 0.001; *n* = 6 animals in each of the groups). Statistical significance in (**G**) was tested using a nested one-way ANOVA followed by a Tukey test for multiple comparisons between pairs of means (* *p* < 0.05, ** *p* < 0.01; *n* = 1595 mini-fascicles in the HT group from *n* = 6 animals, *n* = 66 mini-fascicles in the FF group from *n* = 6 animals, and *n* = 1628 mini-fascicles in the SC-FF group from *n* = 6 animals).

IF using antibodies against GLUT1 and TUBB3 revealed the distribution of perineurial cells (PNCs) and their spatial relationship to axons (Figure 5). The normal distribution of GLUT1-positive PNCs was observed within the perineurium surrounding the proximal (image representative of all groups) and distal nerve segments (Figure 5A,B’’,C’’,D’’,E’’), and the AG at the mid-implant level (Figure 5B’). This arrangement was not observed in the tissue cables in the HT, FF, and SC-FF groups, which all demonstrated varying numbers of mini-fascicles, albeit of different sizes, ensheathed by thin PNC layers (Figure 5C,C’,D,D’,E,E’). The regenerated tissue cable found within the HT was entirely made up of such mini-fascicles of varying sizes (Figure 5C,C’). Only a few axon profiles were not surrounded by PNCs. In the FF and SC-FF groups, mini-fascicles were found around the core, which remained negative for GLUT1 and TUBB3 (Figure 5D,D’,E,E’). In the FF group, many axons lacked ensheathment by PNCs, whereas this was observed only sporadically in the SC-FF group. Interestingly, the SC-FF group demonstrated the most pronounced formation of perineurial ensheathment at the mid-implant level compared with both the HT and FF groups, in some cases even resulting in complete envelopment of the core by PNCs. The endothelial lining of scattered blood vessels at the mid-implant level in the AG and in the distal nerve segments of all groups was also found, with variable intensity, to be GLUT1-positive (Figure 5B’,B’’,D’’,E’’, not shown for HT here but compare Figure S2C’’). No such blood vessels were observed in the HT, FF, or SC-FF implants at the mid-implant level (Figure 5C,C’,D,D’,E,E’). Quantification of the number of GLUT1-positive pixels at the mid-implant level (Figure 5F) revealed significantly higher values in both the HT and SC-FF groups (805,317 ± 105,787 and 817,455 ± 76,978, respectively) than in the FF group (66,556 ± 20,684). Mini-fascicle CSA analysis (Figure 5G) revealed that mini-fascicles were significantly larger in the SC-FF group (228.56 ± 9.34 µm^2^) compared with both the HT and FF groups (104.51 ± 3.68 µm^2^ and 85.53 ± 12.42 µm^2^, respectively), which did not differ significantly from each other.

Blood vessel architecture visualization and angiogenesis assessment was performed by IF with antibodies directed against RECA1 and GLUT1 (Figure S2). Blood vessels in the proximal and distal segments of all groups and within the AG at the mid-implant level expressed both markers. At the mid-implant level, this was not observed in the HT, FF, and SC-FF groups, whose blood vessels practically lacked GLUT1 expression. Furthermore, the cell-dense cores observed in the FF and SC-FF groups were virtually devoid of blood vessels.

**Figure 6:**
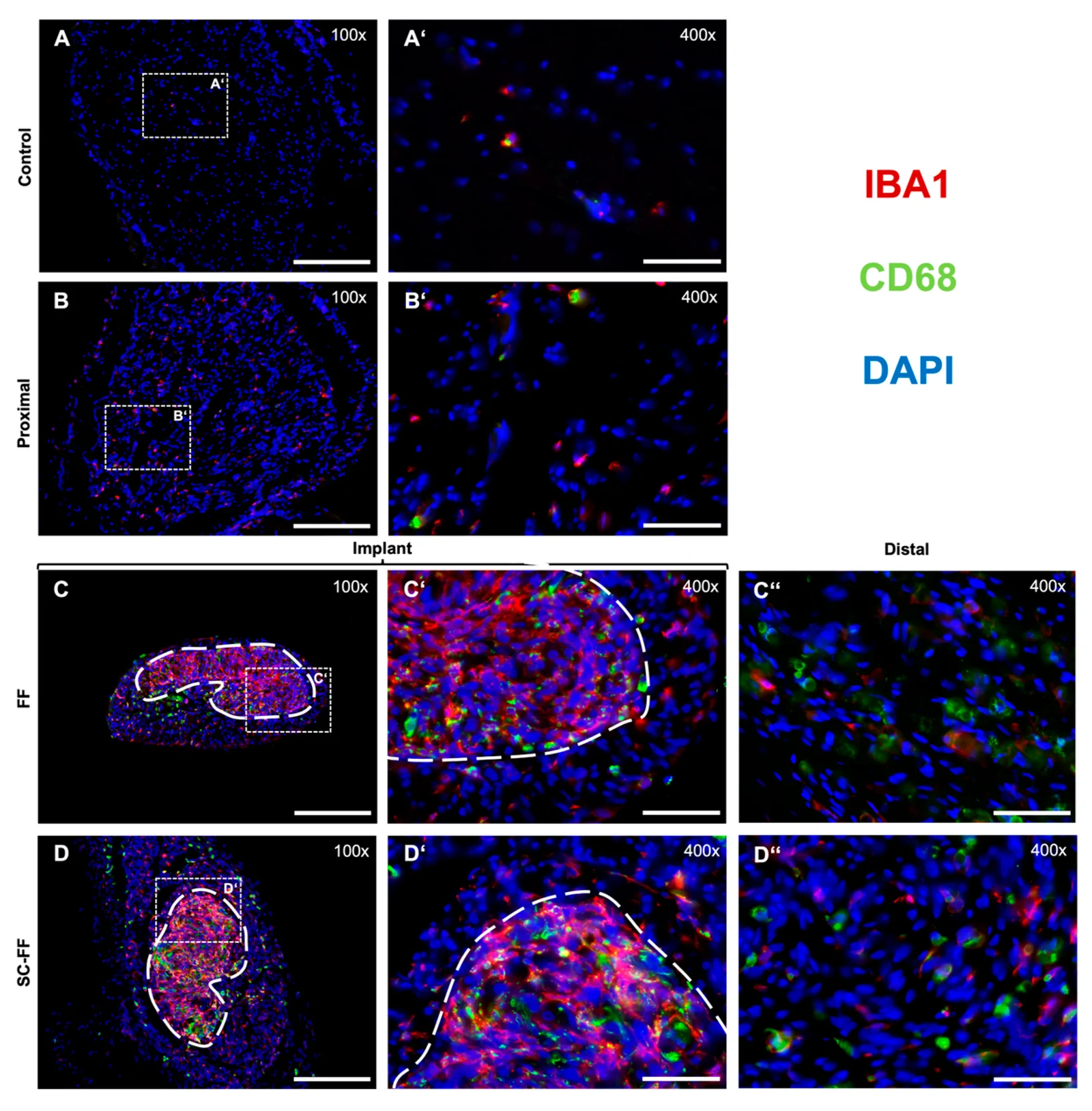
Macrophage-dense cores at the mid-implant levels of the FF and SC-FF groups (cross-sections). (**A**–**B’**) IF for ionized calcium-binding adapter molecule 1 (IBA1, red) and cluster of differentiation 68 (CD68, green) shows a lower macrophage density in the non-lesioned control nerve (**A**,**A’**) compared with the representative proximal nerve segment of an operated animal (**B**,**B’**). IBA1 labels these cells more strongly than CD68. Notably, the overall cell density in the proximal segment of the operated animal is also higher (DAPI, nuclei, blue). (**C**,**C’**,**D**,**D’**) IF reveals that the cores observed in both the FF and SC-FF groups mainly consist of macrophages, with IBA1 again being a more sensitive marker than CD68 ((**C**,**D**), with higher magnifications in (**C’**,**D’**); cores indicated by dashed line). Around the cores, density and distribution of IBA1- and CD68-positive cells resemble the proximal nerve of operated animals. (**C’’**,**D’’**) In the distal nerve segments in the FF and SC-FF groups, numerous IBA1- and CD68-positive cells are also present, and here CD68 appears to be the more sensitive marker for these cells (**C’’**,**D’’**). Scale bars: (**A**–**D**) 200 µm; others 50 µm. Magnification is indicated in the upper right corner for all images. Dashed boxes in the low-power images indicate the region shown at higher magnification in the corresponding high-power images.

IF using antibodies against the macrophage-associated antigens IBA1 and CD68 was performed to characterize the composition of the cell-dense cores at the centers of the FF and SC-FF implants (Figure 6). Control nerves (Figure 6A,A’) and proximal nerve segments of operated animals (Figure 6B,B’) demonstrated the presence of macrophages with fine, scattered processes, which was less accentuated for the non-lesioned controls. Note the overall higher cell density in the proximal nerve segment compared to the control nerve. Interestingly, IBA1 proved to be a more sensitive marker for these cells, whereas only a few CD68-positive profiles were detected, most of which co-expressed IBA1. The IBA1-immunoreactive macrophages, however, were largely CD68-negative. Strikingly, both the FF and SC-FF groups showed a strong immunoreactivity for IBA1 within the cores, with numerous cells demonstrating additional CD68-positivity (Figure 6C,C’,D,D’). Notably, the regenerated mini-fascicles surrounding the cores contained IBA1- and CD68-positive cells at a density comparable to that of the proximal nerve segments in the operated animals. Interestingly, more solely CD68-positive and fewer IBA1-immunoreactive cells were found within the distal nerve segments, making CD68 the predominant marker of macrophages here (Figure 6C’’,D’’).

**Figure 7:**
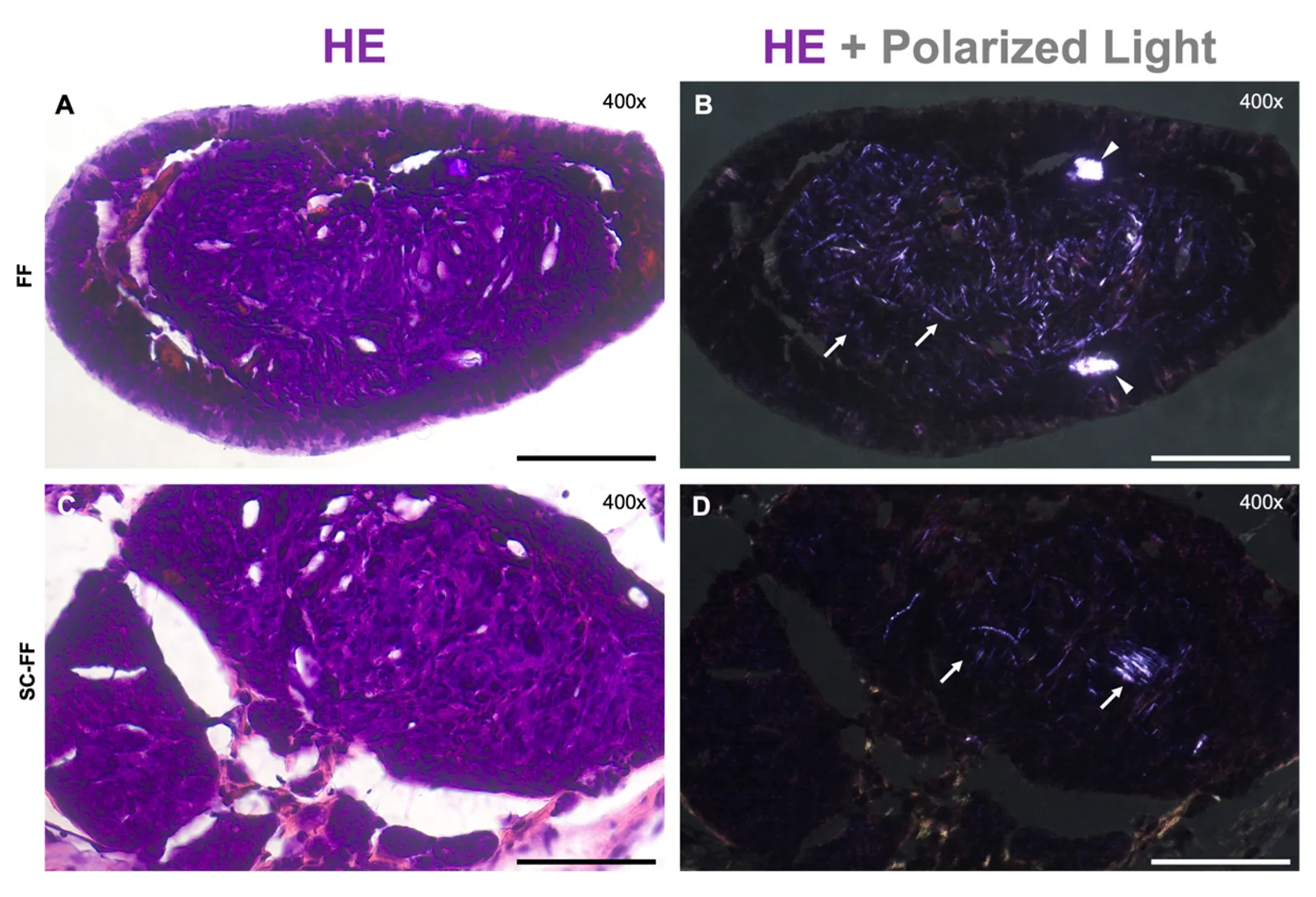
Hematoxylin/eosin stains of the core regions in the FF and SC-FF groups (cross-sections). Conventional brightfield microscopy of the core regions observed in the FF (**A**) and SC-FF groups (**C**). Polarized light microscopy reveals that birefringent nanofibers (arrows) remain present 12 weeks after implantation in both the FF (**B**) and SC-FF implants (**D**), embedded deep within the cores in both groups. Arrowheads in (**B**) indicate suture material. Scale bars: 100 µm. Magnification is indicated in the upper right corner for all images. To facilitate visualization of the PCL nanofibers, color saturation in (**B**,**D**) was reduced to 33%.

Polarized light microscopy of HE-stained FF and SC-FF transverse sections at the mid-implant level unveiled the persistent presence of birefringent PCL nanofibers located deep within the core regions (Figure 7). While HE-stained specimens supported the observation of a cell-dense infiltration of the implant centers as already revealed by IF, no clear formation of core-associated foreign body giant cells (FBGCs) was observed. In contrast, these FBGCs were repeatedly observed in close proximity to the suture material used during surgeries.

Additional IF using antibodies directed against IBA1, P4Hβ and SMA was performed to further characterize the composition of the cell-dense cores seen in the FF and SC-FF groups. While P4Hβ IF revealed the presence of collagen-synthesizing, fibroblast-like cells in the cores of both groups intermingling with the macrophage population (Figure S3), immunoreactivity for SMA was limited to blood vessels that were located mainly around but not within the cores (compare also RECA1 IF results as described above). No colocalization with cells within the cores was observed (Figure S4).

Taken together, the observed infiltration of the FF and SC-FF implants by macrophages and fibroblast-like cells is indicative of a host response to scaffold components in both groups. While characteristics of the later stages of the foreign body response (FBR)—such as prominent FBGC formation and myofibroblast infiltration [28,29,30]—are missing, the absence of both SCs and regenerated axons suggests that this host response does not seem to be pro-regenerative.

Based on these observations, material derived from additional FF and SC-FF animals that had been finalized after two weeks was analyzed to gain further insight into the dynamics of core formation (Figure S5). In the FF group, darkfield microscopy with DAPI counterstaining revealed the expected stacked arrangement of PCL nanofiber arrays within the relatively well-preserved fibrin hydrogel matrix that filled most of the inner lumen of the surrounding collagen HT. The overall cell density within the fibrin hydrogel, however, remained relatively low. In contrast, advanced degeneration of the fibrin hydrogel was observed in the SC-FF group, with the PCL nanofibers coalescing in the center of the fibrin hydrogel matrix, which only occupied a relatively small portion of the lumen of the surrounding collagen HT. In comparison with the fibrin hydrogel seen in the FF group, the cell density within the fibrin hydrogel was much higher in the SC-FF group. It is impossible, however, to determine the extent to which this observation can be attributed to the presence of encapsulated Schwann cells following conduit implantation or to cell invasion after scaffold implantation.

## 4. Discussion

### 4.1. Rationale for Scaffold Design

Here, we evaluated a novel biomimetic scaffold for peripheral nerve repair in a 15 mm rat sciatic nerve resection model, representing a critical-size defect length for HTs. Prefabricated collagen HTs [20,21,22] were used as the base scaffold as they are clinically approved under defined conditions [11]. However, they are known to fail in gaps exceeding 10 mm in rats [17] and 30 mm in humans [10,16], which can be largely attributed to an inadequate formation of a fibrin cable that bridges the defect [18,19]. Therefore, pre-loading the conduit lumen with a fibrin hydrogel was intended to support endogenous fibrin cable formation and provide a permissive matrix for regenerative cell migration. Fibrin has been widely used as a luminal filler in nerve guides, including SC- and growth factor-loaded PCL conduits [31]. Incorporation of guidance cues—either via grooved inner walls [32,33], oriented microchannels [34,35], or aligned luminal fillers [36]—mimics nerve anisotropy and directs regenerative cell migration. To exploit the entire conduit cross-section, stacked arrays of highly aligned PCL nanofibers were embedded into the fibrin hydrogel. Such nanofibers can recapitulate aspects of extracellular matrix topography and induce cell alignment as well as directional migration [37]. Our previous work showed that SCs embedded in similar fibrin hydrogels rapidly redistribute onto aligned nanofibers and extend processes [26], resembling SC alignment in bands of Büngner. Fiber-induced SC alignment has been linked to polarized L1 expression [38] and myelinating SC phenotypes [39,40], indicating that oriented fibers affect both morphology and function.

SCs are key players in nerve regeneration. Following injury, they adopt a c-Jun-dependent repair phenotype, proliferate, and orchestrate regeneration through controlled breakdown of the blood–nerve barrier (BNB) to permit immune cell invasion, clearance of debris, formation of bands of Büngner to guide regenerating axons across the lesion site, and subsequent re-myelination [19,41,42]. Seeding conduits with SCs consistently improves regeneration [43,44,45], reflecting the same key mechanisms that underlie the success of autografts, where preserved endoneurial tubes and repair-competent SCs are delivered to the lesion site [10,11,13]. Accordingly, we evaluated the pre-seeding of our fibrin hydrogel with isogenic SCs to combine physical guidance with the immediate delivery of repair-competent cells. Mechanical testing showed that nanofiber incorporation only slightly increased the fibrin hydrogel’s elastic modulus, suggesting that the mechanical properties of the environment remained within a range compatible with SC repair responses, as excessive stiffness is associated with impaired SC function [46].

### 4.2. Functional Outcome Measures and Retrograde Tracing

Functional recovery during and at the end of the 12-week follow-up period was evaluated by hind-paw grip strength, SSI and muscle weight loss. This was complemented by retrograde tracing of motor and sensory axons that had crossed the lesion site, resulting in labelling of respective neuronal cell bodies in the ventral horn of the spinal cord and DRGs, respectively. Collectively, these data revealed that the AG provided the best support for functional recovery, followed by the SC-FF, HT and FF implants, although many of the observed differences were not statistically significant. In this context, the results of the retrograde tracing analysis of motor neurons, in particular, should be considered preliminary and interpreted with caution, as the number of animals in the LO and AG groups was insufficient for statistical analysis. Notably, while a plateau was found for the SSI after about nine weeks, grip strength in the AG, SC-FF, and HT groups was still improving at 12 weeks, which is consistent with reports that even 16 weeks may be insufficient for maximal recovery after rat sciatic nerve repair in a comparable model [47]. According to Gordon et al., it takes approximately ten weeks for axons to bridge both suture sites in a 15 mm resection injury of the rat sciatic nerve [48]. This would leave only another two weeks for axons to regenerate the entire length of the nerve branches distal to the trifurcation and establish neuromuscular junctions with the target musculature. This could also explain why no significant differences in muscle weight loss were observed between the AG, SC-FF, and HT implants, whereas differences in grip strength were detected, most likely reflecting contributions from more distal muscles that had not yet been re-innervated. Similarly, the delayed innervation of smaller and more distal muscles in all groups might also explain why no differences were observed for SSI performance.

### 4.3. Macroscopic and Histological Findings

Macroscopic findings mirrored functional outcomes: Tissue cables with CSAs comparable to control nerves were only observed in the AG group, whereas the FF group frequently exhibited only small-diameter tissue bridges. In both the FF and SC-FF groups, histology revealed a well-demarcated, cell-dense core within the conduit, composed mainly of CD68- and IBA1-positive macrophages with intermingling P4Hβ-positive fibroblast-like cells, indicating a host response against scaffold components. Polarized light microscopy revealed persistent, often clustered PCL nanofibers within these core regions. Such cores constituted a “dead space” largely devoid of SCs, axons, PNCs, and blood vessels, interfering with the regenerative processes within the tissue bridge and indicating poor tissue integration of the scaffolds.

Quantification of the number of TUBB3-positive pixels both at the mid-implant level and distally revealed the same trends as those observed for behavioral analyses and retrograde tracing—the AG group outperformed the others, followed by the SC-FF, HT, and FF groups. SC delivery enhanced the regenerative potential of the SC-FF scaffold, even in the presence of the core, consistent with the earlier onset of measurable grip-strength recovery in the SC-FF group compared with the HT group. Generally, axons within the distal nerve segment appeared thinner than proximally, consistent with ongoing sprouting and indicating—also in line with functional outcome measures—that maximal regeneration had not yet been reached [48], resulting in discrepancies between distal axon density and functional recovery. As distal axon density reflects successful crossing of both suture sites and reintegration into the distal nerve architecture, however, it correlated better with functional outcomes than the mid-implant counts.

All intervention groups except the AG group showed a growth pattern resembling mini-fascicles, with axon-SC bundles being encased by thin GLUT1-positive perineurial sheaths [49,50,51,52]. At the mid-implant level, the GLUT1 signal was higher in the HT and SC-FF implants than in the FF implants, suggesting that core formation in the FF group impeded PNC migration. In the SC-FF group, SC delivery may have secondarily enhanced PNC recruitment, possibly via Dhh/PTCH1 signaling [53], producing significantly larger mini-fascicles than those seen in the other groups. Axons outside mini-fascicles were also observed, particularly in the FF implants, although these fibers lacked appropriate SC and perineurial support and are therefore unlikely to support optimal long-term function.

### 4.4. Macrophages, Host Response and Neovascularization

Macrophages are indispensable for nerve repair, contributing to myelin clearance, vascular endothelial growth factor (VEGF)-dependent neovascularization, and confinement of SCs and axons within the nerve bridge via Slit3/Robo1 signaling [19,41,54,55,56]. As described by Ydens et al., the main macrophage response to PNI is driven by monocyte-derived invading macrophages and these cells express the highest VEGF levels [57]. Outside the nervous system, VEGF expression in macrophages is mainly associated with the M1 phenotype, which suggests that some extent of M1 response is required for proper nerve bridge vascularization, whereas M2 macrophages seem to be mainly involved in blood vessel maturation [58]. The formation of a macrophage-rich core observed in the FF and SC-FF implants, however, is unlikely to reflect the expected infiltration of these cells as part of the normal regenerative response following PNI but rather a maladaptive host response to the scaffold components. This becomes particularly clear when compared with the HT implants, where no such macrophage-rich core formation was observed at 12 weeks in the absence of the luminal fillers (i.e., the PCL nanofiber-loaded fibrin hydrogel ± SCs) under otherwise identical conditions.

Xenogenic fibrin hydrogels have repeatedly been used in rat nerve repair without evidence of immunological incompatibility [23,31,59,60,61,62]. Wolbank et al. have reported the complete degradation of subcutaneously implanted fibrin hydrogels in nude mice after about 16 days [63]. In contrast, our own data show that—in the absence of encapsulated SCs—the fibrin hydrogels remain relatively well preserved after an implantation time of two weeks. The apparently slower degradation kinetics in comparison to the results obtained by Wolbank et al. could be due to the incubation with tranexamic acid-containing medium prior to scaffold implantation, delaying fibrinolysis. In the presence of encapsulated SCs, however, we saw indications of accelerated fibrin hydrogel degradation, resulting in coalescing of the embedded PCL nanofibers within the center of the conduit, highlighting that degradation of the different scaffold components was not well synchronized. Since our earlier in vitro study [26] only involved incubation times of SCs within the fibrin hydrogels of up to seven days and in the continuous presence of tranexamic acid, it is not surprising that signs of fibrin hydrogel degradation were not observed previously. In the literature, it has been well established that SCs release fibrinolytic substances such as tissue plasminogen activator and urokinase, and that degradation of the naturally forming fibrin cable is necessary, as prolonged fibrin deposition may exacerbate inflammatory axonal degeneration [64,65,66].

Although PCL has a well-established record of biocompatibility across numerous applications [24,25], a number of studies have described PCL-associated host responses, including FBRs with formation of FBGCs and fibrosis, in a range of tissues—including nervous tissue [67,68,69,70,71,72]. Given our observation that the PCL nanofibers could still be identified within the cell-dense cores that had formed within the FF and SC-FF implants, it seems likely that the host response was caused by the PCL nanofibers rather than the fibrin hydrogel. In a previous investigation, Kriebel et al. investigated the in vivo performance of a scaffold design very similar to ours, for which electrospun nanofibers made of either PCL (their group G1) or a collagen-PCL blend (their group G2) were embedded within a non-crosslinked gelatin matrix inside identical collagen HTs [20]. Although no fibrin hydrogel was used at the time, the authors reported that “Cross-sections of several regenerated nerves showed in eight of 14 samples of groups G1 and G2 some tissue in the centre, which was devoid of axons and Schwann cells but filled with S100^-^/βIII-tubulin^-^ cells”, leaving the use of (collagen-blended) PCL nanofibers the only common denominator between the two studies. However, as no colocalization of the SC- and axon-free area with persisting PCL nanofibers was demonstrated, this association remains somewhat speculative.

The host response to medical-grade PCL following soft tissue implantation in a porcine model was recently characterized in detail by Klein et al. over the course of one year [72]. In their study, M1 macrophages and FBGCs were consistently observed adjacent to the PCL fibers and surrounded by a ring-like arrangement of M2 macrophages which progressively intermingled with (myo-)fibroblasts, collagen fibers and blood vessels at increasing distances from the implant. While an M1 macrophage response seems to be initially required for successful nerve repair (see above), the fact that these cells were constantly present in association with the PCL fibers over the course of one year—as reported by Klein et al.—raises concerns as to whether this pro-inflammatory environment could interfere with nerve regeneration in the long term, as seen in our scaffolds. Following implantation of a foreign material into the body, it has been reported that macrophage and lymphocyte infiltration usually peak between two and five weeks, while a potential foreign body granuloma forms later [28]. With regard to the above-mentioned timeline for regeneration across a 15 mm rat sciatic nerve resection injury [48], both nerve regeneration and host response against the scaffold components likely overlap at least temporarily, which—considering the long-lasting M1 response induced by PCL fibers—poses a risk for detrimental interference. Klopfleisch compared the macrophage response to degradable and non-degradable biomaterials, highlighting that degradable biomaterials ideally induce an early M1 presence and late M2 dominance [29], as degradation of the material can be mainly attributed to M1 macrophages and subsequent tissue remodeling, in contrast to M2 macrophages. Given the slow biodegradability of PCL, this is alarmingly consistent with the observations of Klein et al., resulting in a long-term M1 response around their filaments [72]. However, given the delicate process of nerve regeneration, which is supposed to occur unimpeded within the lumen of our implant, the intended response to a non-degradable biomaterial [29]—minimal initial M1 response and early but restricted M2 dominance—may represent a more favorable response, as it may minimize interference between nerve regeneration and biomaterial degradation.

The PCL filaments studied by Klein et al. had a diameter of around 350 µm [72], which might affect host response and degradation behavior in vivo in comparison with nanoscale fibers. Dias et al. and Bölgen et al. studied the in vivo degradation of electrospun PCL nanofiber mats in mice and rats, respectively [73,74], although it was not specified whether medical-grade PCL was used. While Bölgen et al. focused more on the material side and reported a weight loss of 27% after 90 days in vivo, Dias et al. focused on the tissue response, highlighting the formation of FBGCs after the same period.

In addition to the filament size, the use of non-medical-grade PCL in the current study is another potentially crucial difference to the study by Klein et al. [72], as the potential presence of endotoxins could be a relevant confounding factor. PCL nanoparticles have been demonstrated to exhibit a high affinity to endotoxins [75] and the low melting point of PCL prevents the use of established inactivation methods, particularly heat treatment [76]. While the presence of endotoxins has been shown to increase the initial inflammatory response to an implanted material, the long-term host response appears to be mostly unaffected [77,78]. To this end, the delayed formation of FBGCs in the presence of endotoxins described by van Putten et al. is consistent with our observation that FBGCs were not a prominent component of the host response against the FF and SC-FF scaffolds—a finding that contrasts with the descriptions provided by Dias et al. [74]. That the scaffold components did not induce a fully mature foreign body granuloma in our study is also supported by the absence of SMA immunoreactivity, a key marker of fibrosis in the context of the FBR [30]. The PCL used in this study, however, was not initially tested for endotoxin contamination, and a subsequent test was not appropriate because the possibility of contamination during handling cannot be ruled out. It is, in consequence, not possible with absolute certainty to attribute the observed host response solely to the polymer itself.

As mentioned above, vascularization of the nerve bridge is a key macrophage-mediated process [55] and Plexin-D1-dependent directed blood vessel ingrowth prevents fibrotic scarring and supports formation of GLUT1-positive stromal tubes that guide regenerating axons and SCs [79]. In our study, blood vessels were abundant near mini-fascicles in the HT and SC-FF implants but sparse in the FF implants, paralleling low GLUT1 signal and poor mini-fascicle formation. Interestingly, the core regions were practically avascular, indicating that macrophages involved in the core formation did not induce sufficient neovascularization. The mid-implant blood vessels in the HT, SC-FF, and FF groups were largely GLUT1-negative, suggesting a disturbance of the BNB, whereas proximal and distal segments and the AG mid-implant regions showed distinctive GLUT1-positive endoneurial blood vessels [80,81,82]. Given the lack of blood vessels within the core and the importance of neovascularization for successful nerve repair [55], enhancing core vascularization (e.g., by VEGF delivery) appears promising but could exacerbate the host response by increasing monocyte influx [83]. Consequently, Dondossola et al. even proposed disrupting this vicious cycle by preventing the accumulation of macrophages and neovascularization through targeted VEGF inhibition [83]. However, as both macrophages and neovascularization are indispensable for nerve repair, broad suppression is likely unsuitable for this very scenario. In turn, more focused approaches—such as targeting the NLRP3 inflammasome—could be investigated, as these have been shown to attenuate the FBR to implanted biomaterials while maintaining angiogenesis [84].

## 5. Conclusions

In a previous study [26], we demonstrated that aligned PCL nanofibers embedded in fibrin hydrogels provide effective topographical guidance in vitro. The present study, however, demonstrates that their in vivo application in the present form is limited by a macrophage-rich host response which competes with the regenerative processes following PNI. Pre-seeding of the fibrin hydrogels with SCs partially overcame these limitations and improved performance in comparison with the non-SC-seeded scaffold but did not achieve the level of regeneration observed in the AG group. Furthermore, our results highlight that the in vivo degradation behavior of different materials used in the same scaffold must be well matched in terms of their intended purposes. We believe that these findings provide crucial insights for the design of future nerve guidance conduits. In light of the desired macrophage responses to degradable and non-degradable materials described by Klopfleisch [29], we advocate for the evaluation of non-degradable materials in nervous tissue repair.

## Figures and Tables

**Table 1:** Overview of primary antibodies used for IF.

Species/ Clonality	Antigen	Dilution	Product Number	Manufacturer
Mouse monoclonal	βIII-tubulin (TUBB3)	1:1000	T8660	Sigma-Aldrich
	rat endothelial cell antigen 1 (RECA1)	1:500	MCA970R	Bio-Rad, Feldkirchen, Germany
	cluster of differentiation 68 (CD68)	1:1000	BM4000S	OriGene, Herford, Germany
	prolyl-4-hydroxylase β (P4Hβ)	1:500	AF5110-1	OriGene
	smooth muscle actin (SMA)	1:1000	M0851	Dako
Rabbit polyclonal	protein S100β (S100)	1:1000	Z0311	Dako
	glucose transporter 1 (GLUT1)	1:500	OAAI00167	Avivasysbio, San Diego, CA, USA
	ionized calcium-binding adapter molecule 1 (IBA1)	1:1000	019-19741	Wako, Neuss, Germany

## Data Availability

The raw data supporting the conclusions of this article will be made available by the authors upon reasonable request.

## Supplementary Materials

The following supporting information can be downloaded at https://www.mdpi.com/article/10.3390/biomimetics11090648/s1, Figure S1: Stepwise manufacturing procedure of fibrin-nanofiber scaffolds; Figure S2: Rat endothelial cell antigen 1 (RECA1) and GLUT1 IF of sciatic nerve explants from all intervention groups (cross-sections); Figure S3: IBA1 and prolyl-4-hydroxylase β (P4Hβ) IF of the core regions seen in the FF and SC-FF groups (cross-sections); Figure S4: IBA1 and smooth muscle actin (SMA) IF of the core regions seen in the FF and SC-FF groups (cross-sections); Figure S5: Darkfield microscopy and DAPI staining of the FF and SC-FF implants at mid-implant level after an implantation period of two weeks (cross-sections); Table S1: Muscle weight loss for each muscle and group, given as mean ± SEM (in % compared with the contralateral healthy side).

## Abbreviations

The following abbreviations are used in this manuscript:

PCL	Poly(ε-caprolactone)
SC	Schwann cell
FBR	Foreign body response
PNI	Peripheral nerve injury
AG	Autograft
HT	Hollow tube
SC-FF	Schwann cell-seeded fibrin-nanofiber scaffold
FF	Fibrin-nanofiber scaffold
LO	Lesion-only control group
DMEM	Dulbecco’s Modified Eagle Medium
FBS	Fetal bovine serum
PLL	Poly-L-lysine
SCGFM	Schwann cell growth factor medium
IF	Immunofluorescence
PBS	Phosphate-buffered saline
SSI	Static sciatic index
PFA	Paraformaldehyde
EDL	Extensor digitorum longus muscle
TA	Tibialis anterior muscle
SOL	Soleus muscle
GC	Gastrocnemius muscle
DRG	Dorsal root ganglion
DAPI	4′,6-diamidino-2-phenylindole
HE	Hematoxylin/eosin
TUBB3	βIII-tubulin
RECA1	Rat endothelial cell antigen 1
CD68	Cluster of differentiation 68
SMA	Smooth muscle actin
S100	Protein S100β
GLUT1	Glucose transporter 1
IBA1	Ionized calcium-binding adapter molecule 1
CSA	Cross-section area
SEM	Standard error of the mean
ANOVA	Analysis of variance
PNC	Perineurial cell
BNB	Blood–nerve barrier
VEGF	Vascular endothelial growth factor
FBGC	Foreign body giant cell
