# Supplementary material for: Host Response Impairs Tissue Integration of a Fibrin Hydrogel Scaffold Containing Poly(ε-caprolactone) Nanofibers for Peripheral Nerve Repair
Source: Biomimetics (Basel). 2026 Sep 9;11(9):648. doi: 10.3390/biomimetics11090648 (PMC13604744; doi:10.3390/biomimetics11090648)
Supplement: Supplementary file 1 [file biomimetics-11-00648-s001.zip › biomimetics-4517689-supplementary.pdf]

Supplementary Material for

Host Response Impairs Tissue Integration of a Fibrin Hydrogel Scaffold Containing Poly(ε-caprolactone) Nanofibers for Peripheral Nerve Repair

Haktan Altinova [1,2,3,*], Dorothee Hodde [1,4], José L. Gerardo-Nava [5,6], Axel Dievernich [7], Lmar Arman [1], Melissa Büchler [8], Andreas Kriebel [9], Jörg Mey [9,10], Hans Clusmann [2], Joachim Weis [1], Gary A. Brook [1,†] and Pascal Achenbach [1,8,11,*]

[1] Institute of Neuropathology, Uniklinik RWTH Aachen, 52074 Aachen, Germany
[2] Department of Neurosurgery, Uniklinik RWTH Aachen, 52074 Aachen, Germany
[3] Division of Medical Assessment, State Office for Health and Social Affairs (Lageso), 10559 Berlin, Germany
[4] LMU University Hospital, LMU Medizin, Ludwig-Maximilians-Universität München, 80337 Munich, Germany
[5] DWI–Leibniz Institute for Interactive Materials, 52074 Aachen, Germany
[6] Institute of Applied Medical Engineering, Department of Advanced Materials for Biomedicine, Uniklinik RWTH Aachen, 52074 Aachen, Germany
[7] FEG Textiltechnik Forschungs-und Entwicklungsgesellschaft mbH, 52070 Aachen, Germany
[8] Department of Neurology, Uniklinik RWTH Aachen, 52074 Aachen, Germany
[9] Institute of Biology II, RWTH Aachen University, 52074 Aachen, Germany
[10] Laboratorio de Regeneración Neural, Hospital Nacional de Parapléjicos/IDISCAM, 45071 Toledo, Spain
[11] Phil and Penny Knight Campus for Accelerating Scientific Impact, University of Oregon, Eugene, OR 97403, USA
* Correspondence: haltinova@ukaachen.de (H.A.); pachenbach@ukaachen.de (P.A.)
† GAB co-supervised the work but passed away during the preparation of this manuscript.

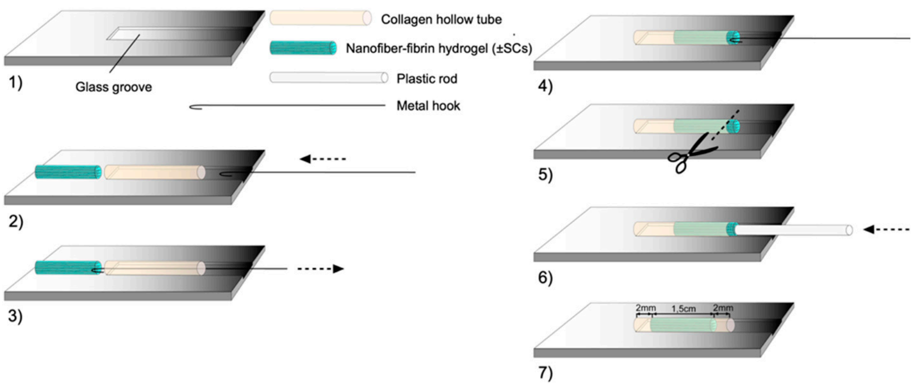

Figure S1. Stepwise manufacturing procedure of fibrin-nanofiber scaffolds. Nanofiber-containing fibrin hydrogel strips (±SCs) were pulled into phosphate-buffered saline (PBS)-soaked, prefabricated collagen type I/III hollow tube conduits using a glass groove and a custom-made metal hook. The end damaged by the hook was then cut off using scissors, and the hydrogel was gently pushed back into the conduit with a blunt plastic rod to avoid further damage. This procedure left a few millimeters of hollow tube conduit protruding from each end of the conduit, allowing for tensionless epineurial suturing to the nerve stumps.

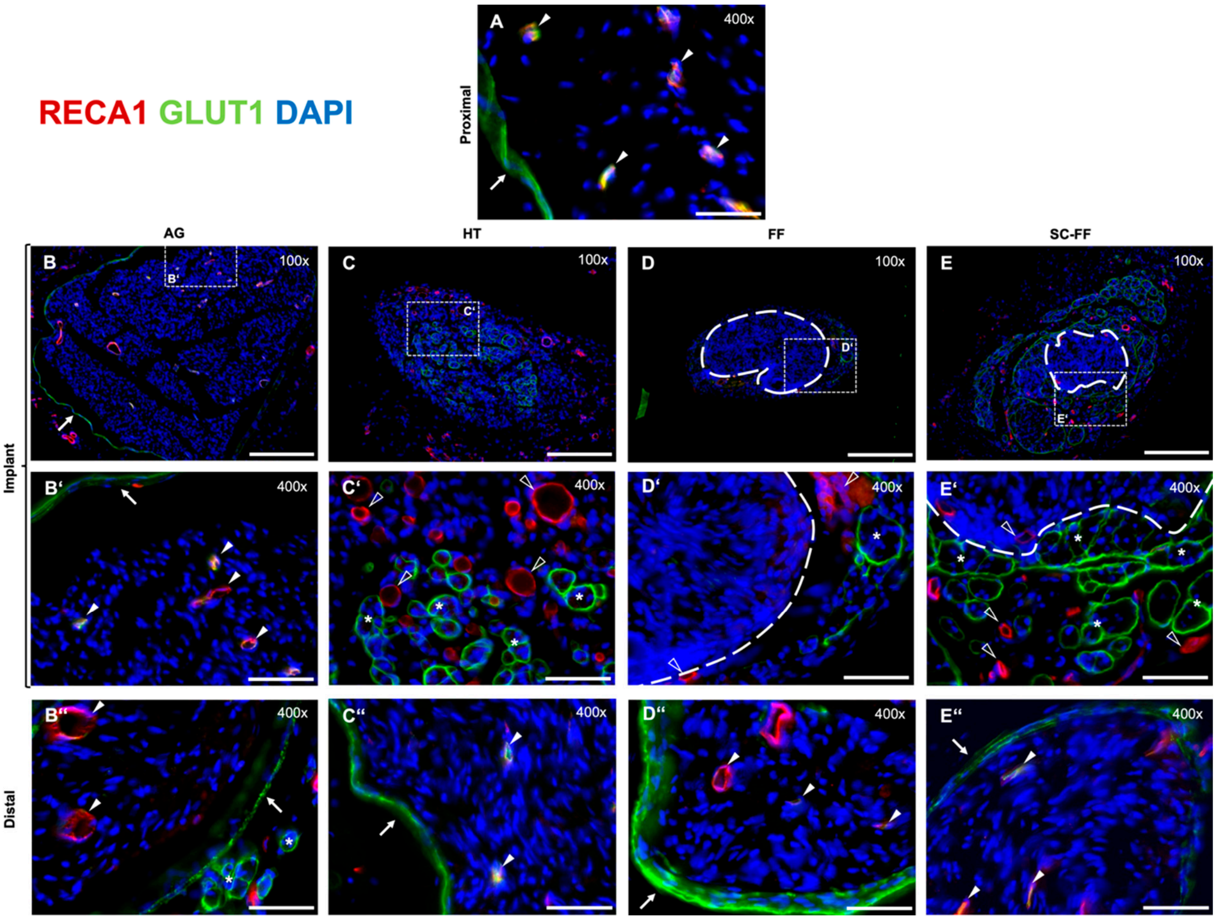

Figure S2. Rat endothelial cell antigen 1 (RECA1) and GLUT1 IF of sciatic nerve explants from all intervention groups (cross-sections). (**A–B″**) Numerous blood vessels (arrowheads) co-expressing RECA1 (red) and GLUT1 (green) are present in the proximal nerve segment (**A**, image representative of all groups), the AG implant (**B**,**B′**) and the respective distal nerve segment (**B″**). The perineurium is indicated by arrows. Individual mini fascicles (asterisks in **B″**) in close proximity to the distal nerve segment suggest some degree of neuroma formation. (**C–C″**) At the mid-implant level of the HT group, numerous RECA1-positive/GLUT1-negative blood vessels (empty arrowheads in **C′**) are found between but usually not within regenerated mini fascicles (asterisks in **C′**). In the distal segment (**C″**), however, blood vessels are located within the fascicles and are RECA1-positive/GLUT1-positive (perineurium indicated by arrow, blood vessels by arrowheads). (**D–D″**) Only sporadic RECA1-positive/GLUT1-negative blood vessels (empty arrowheads in **D′**) are present in the FF group around the core (dashed lines in **D**,**D′**), mainly at its outer edge. The remainder of the core is avascular. Most blood vessels are found close by but not within regenerated mini fascicles (asterisk in **D′**). Distally (**D″**), blood vessels are located within nerve fascicles and co-express RECA1 and GLUT1 (perineurium is indicated by arrow, blood vessels are indicated by arrowheads). (**E–E″**) In the SC-FF group, similar to the FF and HT groups, RECA1-positive/GLUT1-negative blood vessels (empty arrowheads in **E′**) are located in between, but generally not within the regenerated mini fascicles (asterisks in **E′**). The core region (dashed lines in **E**,**E′**) contains only a few blood vessels at its outer edge. In the distal segment (**E″**), blood vessels are located within fascicles and are RECA1-positive/GLUT1-positive (perineurium is indicated by arrow, blood vessels are indicated by arrow heads). Scale bars: (**B**,**C**,**D**,**E**) 200 μm; others 50 μm. Magnification is indicated in the upper right corner for all images. Dashed boxes in the low-power images indicate the region shown at higher magnification in the corresponding high-power images.

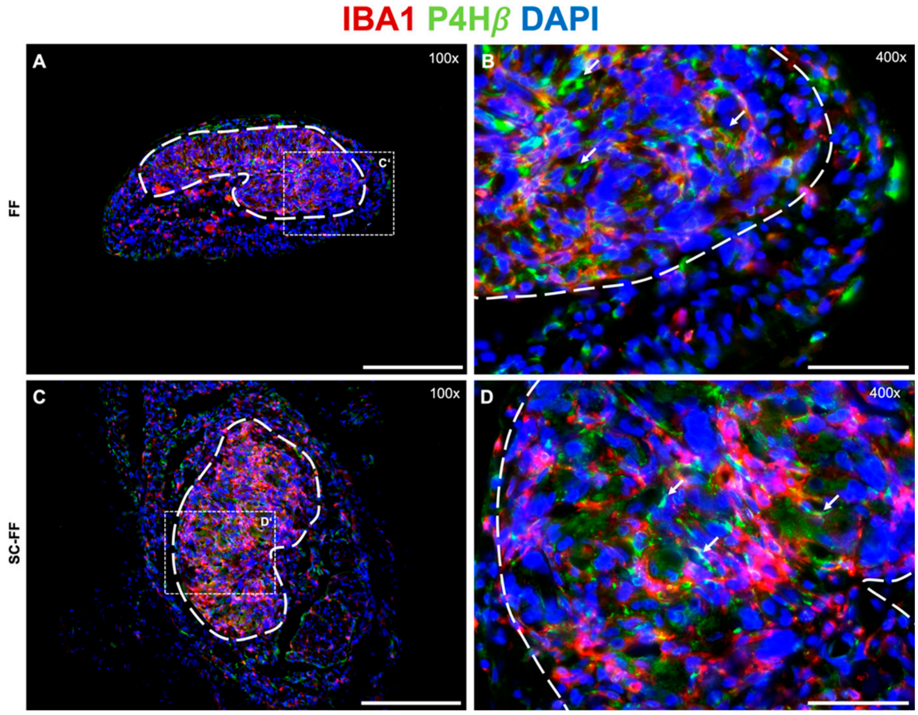

Figure S3. IBA1 and prolyl-4-hydroxylase β (P4Hβ) IF of the core regions seen in the FF and SC-FF groups (cross-sections). The core regions are outlined by dashed lines in all images. Accumulations of P4Hβ-positive fibroblast-like cells (green) are found in the core regions of both the FF (**A**, higher magnification in **B**, here indicated by arrows) and SC-FF implants (**C**, higher magnification in **D**, here indicated by arrows). Around the core regions, these cells are also present but in comparably lower density. Inside the cores, such fibroblast-like cells seem to be intermingling with IBA1-positive macrophages (red). Scale bars: (**A**,**C**) 200 μm; (**B**,**D**) 50 μm. Magnification is indicated in the upper right corner for all images. Dashed boxes in the low-power images indicate the region shown at higher magnification in the corresponding high-power images.

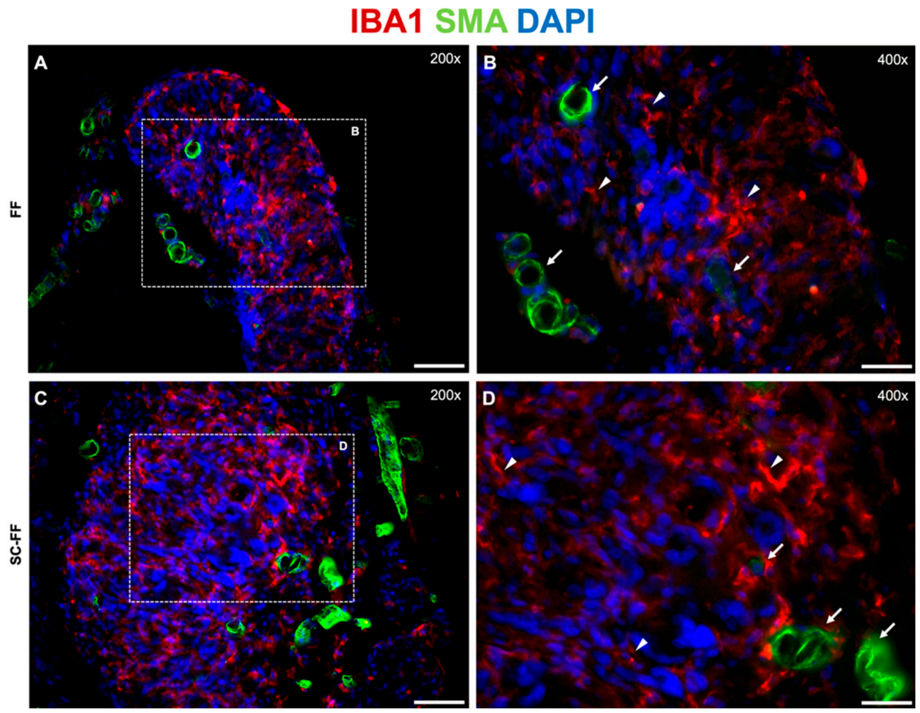

Figure S4. IBA1 and smooth muscle actin (SMA) IF of the core regions seen in the FF and SC-FF groups (cross-sections). Accumulations of IBA1-positive macrophages (red) can be found in the core regions of both the FF (**A**, higher magnification in **B**) and SC-FF groups (**C**, higher magnification in **D**). SMA (green) strongly labels smooth muscle cells in blood vessel walls (arrows in **B,D**) but does not colocalize with the macrophages present within the cores (arrowheads in **B,D**). Scale bars: (**A,C**) 50 µm; (**B,D**) 25 µm. Magnification is indicated in the upper right corner for all images. Dashed boxes in the low-power images indicate the region shown at higher magnification in the corresponding high-power images.

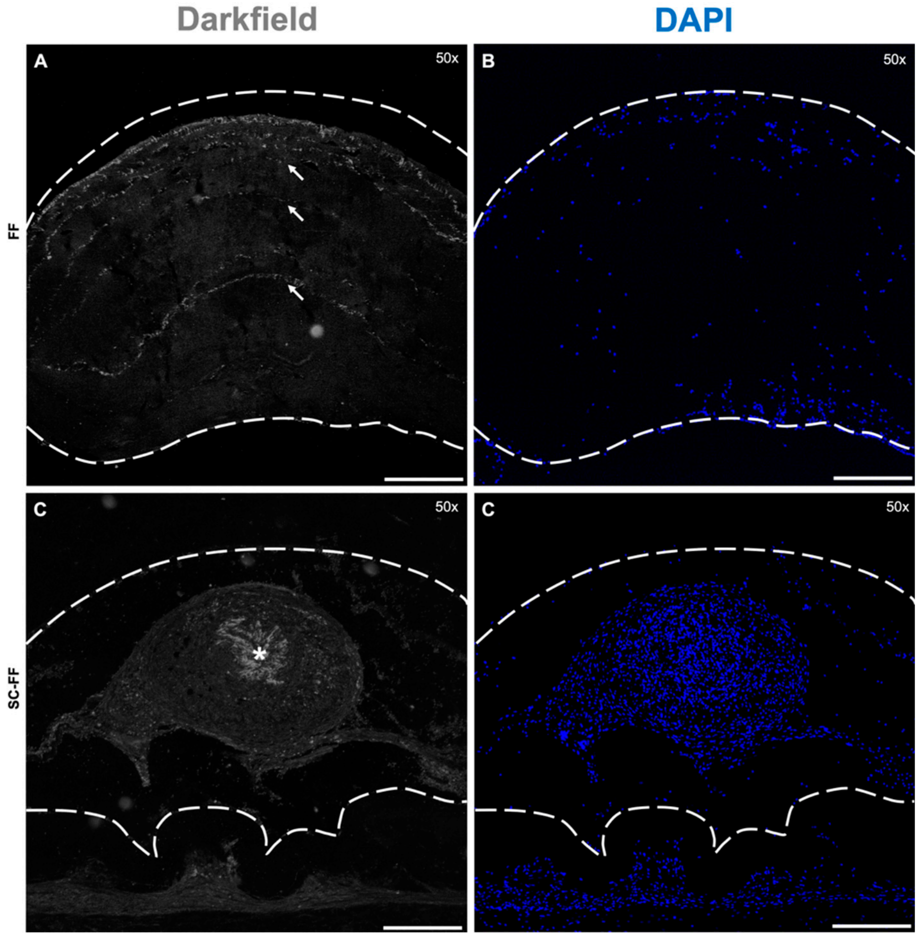

Figure S5. Darkfield microscopy and DAPI staining of the FF and SC-FF implants at mid-implant level after an implantation period of two weeks (cross-sections). The inner lumina of the outer collagen hollow tube conduits are indicated by dashed lines in all images. For the FF implant, darkfield microscopy (**A**) demonstrates the intended layered arrangement of PCL nanofiber arrays within the fibrin hydrogel (arrows). The fibrin hydrogel appears to be well-preserved, filling almost the entire lumen of the collagen hollow tube conduit. DAPI staining (**B**) reveals a relatively low cell density within and around the fibrin hydrogel. For the SC-FF implant, in contrast, coalescence of PCL nanofibers (asterisk) in the center of the fibrin hydrogel can be seen using darkfield microscopy (**C**). In addition, the hydrogel only occupies a relatively small portion of the lumen of the collagen hollow tube conduit, which is indicative of advanced degradation. This is accompanied by a relatively higher cell density within the fibrin hydrogel as revealed by DAPI staining (**D**). It cannot be determined to what extent this high cell density can be attributed to SC encapsulation within the hydrogel at the time of implantation or to secondary cell invasion after implantation. Scale bars: 250 µm. Magnification is indicated in the upper right corner for all images.

Table S1. Muscle weight loss for each muscle and group, given as mean ± SEM (in % compared with the contralateral healthy side).

Muscle	LO	AG	HT	FF	SC-FF
EDL	70.87 ± 0.68	13.94 ± 2.74	48.64 ± 8.45	71.8 ± 1.17	41.31 ± 5.6
TA	75.76 ± 0.95	27.06 ± 1.46	53.03 ± 8.85	78.45 ± 0.54	51.45 ± 6.81
GC	82.7 ± 0.86	44.05 ± 2.32	68.36 ± 5.39	82.12 ± 0.3	61.11 ± 5.39
SOL	65.31 ± 3.12	30.5 ± 5.94	57.6 ± 7.57	74.31 ± 1.24	55.46 ± 5.64
